# Epistemic Beliefs in Science—A Systematic Integration of Evidence From Multiple Studies

**DOI:** 10.1007/s10648-022-09661-w

**Published:** 2022-02-12

**Authors:** Julia Schiefer, Peter A. Edelsbrunner, Andrea Bernholt, Nele Kampa, Andreas Nehring

**Affiliations:** 1 Hector Research Institute of Education Sciences and Psychology, University of Tübingen, Walter-Simon-Str. 12, 72072 Tübingen, Germany; 2 Department of Humanities, Social and Political Sciences, ETH Zurich, Clausiusstr. 59, 8092 Zurich, Switzerland; 3 Leibniz Institute for Science and Mathematics Education, Kiel, Germany; 4 University College of Teacher Education Tyrol, Innsbruck, Austria; 5 University of Hannover, Hannover, Germany

**Keywords:** Epistemic beliefs, Development, Primary school, Secondary school, Latent profile analysis

## Abstract

Recent research has integrated developmental and dimensional perspectives on epistemic beliefs by implementing an approach in which profiles of learners’ epistemic beliefs are modeled across multiple dimensions. Variability in study characteristics has impeded the comparison of profiles of epistemic beliefs and their relations with external variables across studies. We examined this comparability by integrating data on epistemic beliefs about the source, certainty, development, and justification of knowledge in science from six studies comprising *N* = 10,932 German students from elementary to upper secondary school. Applying latent profile analyses to these data, we found that profiles of epistemic beliefs that were previously conceptualized were robust across multiple samples. We found indications that profiles of epistemic beliefs homogenize over the course of students’ education, are related to school tracking, and demonstrate robust relations with students’ personal characteristics and socioeconomic background. We discuss implications for the theory, assessment, and education of epistemic beliefs.

**Supplementary Information:**

The online version contains supplementary material available at 10.1007/s10648-022-09661-w.

## Introduction

Promoting student achievement in STEM (Science, Technology, Engineering, and Mathematics) is a cornerstone of current educational research and practice (OECD, 2016). To understand the fundamental elements of our world and to be able to participate in socio-scientific discussions in everyday life, it is essential not only to have knowledge and skills in STEM but also to understand the genesis and development of scientific knowledge. Such conceptions about the nature of knowledge and knowing, called epistemic beliefs (Mason & Bromme, 2010), are considered important for students’ learning and understanding of science (Elby et al., 2016; Greene et al., 2018).

In an integrated approach to the modeling of epistemic beliefs, earlier developmental and dimensional models have been integrated (Greene et al., 2008). In research following this approach, multiple dimensions of epistemic beliefs (e.g., about the source & the certainty of knowledge) are assessed with Likert-scale questionnaires. Person-centered statistical models such as latent profile analysis are then applied to the resulting data, to carve out profiles of epistemic beliefs (J. A. Chen, 2012; L. E. Ferguson & Bråten, 2013; Greene et al., 2010; Kampa et al., 2016). The resulting profiles describe developmental stages, represented by subgroups of learners who differ systematically from each other in the strength of some or all dimensions of epistemic beliefs. This integrated approach has been implemented in various studies, and it has enabled first insights into the development of epistemic beliefs and their relations to student variables such as school achievement, motivation, and socioeconomic status (e.g., J. A. Chen, 2012; Kampa et al., 2016).

It is difficult to gauge how the results from different studies that have adopted this approach compare with, are consistent with, extend, or replicate each other. This impedes theoretical development regarding the validity of theorized developmental stages of epistemic beliefs and their relations with external student variables. The goal of the present study is to integrate and compare the findings from these studies. We conduct a systematic re-analysis and integration of the results from six studies that have employed Likert-scale questionnaires covering four dimensions of epistemic beliefs in science (Conley et al., 2004). We investigate the robustness and comparability of profiles of epistemic beliefs, and their relations to individual student characteristics, across these studies. We undertake this integration of evidence to examine whether theorized developmental stages of students’ epistemic beliefs occur and show similarities and differences across different samples, age groups, and academic backgrounds, in order to further our understanding about the development of epistemic beliefs and their relations to external student characteristics.

### Epistemic Beliefs in Science: Developmental and Dimensional Models

Epistemic beliefs are individual beliefs about the *nature of knowledge* (beliefs about what knowledge is) and the *nature of knowing* (beliefs about knowledge acquisition; Hofer & Pintrich, 1997; Lederman, 2007). They are part of broader research on epistemic cognition, which is “the thinking people do about what and how they know” (Sandoval et al., 2016, p. 457)*.* In contrast to models of epistemic cognition that describe epistemic aims, values, and processes for achieving epistemic aims and focus on their situativity and normative aspects (Barzilai & Chinn, 2018; Chinn et al., 2011, 2014), models of epistemic beliefs focus on describing individuals’ beliefs about the structure, sources, and development of knowledge and knowing. These beliefs show some stability across contexts, yet can also differ across domains and topics (Merk et al., 2018; Muis et al., 2006; Sandoval et al., 2016).

Two major lines of research on epistemic beliefs that build the basis for the present study have dominated earlier research. The first line of research followed a developmental view of individuals’ epistemic beliefs (e.g., Kuhn et al., 2000; Perry, 1970). The developmental view differentiates the qualitative stages that individuals commonly go through in the development of their epistemic beliefs. Building on pioneering work of Perry (1970), the central model for from a qualitative perspective was established by Kuhn and Weinstock (2002), who defined and described different stages that occur over the course of schooling: *realist*, *absolutist*, *multiplist*, and *evaluativist*. The authors suggested that preschoolers can be described as *realists* (assuming that assertions are copies of an external reality and that knowledge comes from external authorities) but already show some epistemic awareness. Children at the elementary school level are described as *absolutists* (judging knowledge as “absolute, certain, non-problematic, right or wrong,” p. 376). Students reach the *multiplist* level (also called *multiplistic*, understanding that knowledge is generated by humans & might be considered as opinion) between middle and late childhood. Finally, at the *evaluativist* level, an individual believes that there are “shared norms of inquiry and knowing, and some positions may be reasonably more supported and sustainable than others” (Mason, 2016, p. 376).

The second earlier line of research focused on different dimensions of epistemic beliefs (Hofer, 2016; Schommer, 1990). Although there is consensus on the existence of multiple more or less independent dimensions of epistemic beliefs (Hofer, 2016), a vivid debate about the specific dimensions of the construct has evolved (see Chinn et al., 2011; Hofer, 2016). In the present study, we refer to the well-known four-dimensional model of epistemic beliefs in science conceptualized by Conley et al. (2004). According to this model, central epistemic beliefs in the domain of science include beliefs about the *certainty*, *development, source*, and *justification of knowledge*. The *certainty* dimension reflects beliefs about the stability of knowledge in the natural sciences. It ranges from beliefs in a high level of certainty about knowledge to stances that include the possibility that scientific knowledge can change and that a variety of answers to complex problems can exist. The *development* dimension is associated with beliefs that recognize science as an evolving discipline. It ranges from the idea that scientific knowledge does not develop to statements that scientific answers are continuously developing (e.g., based on new evidence). The *source* dimension addresses beliefs about the knowledge that resides in external authorities. Stances range from strict beliefs in authorities (e.g., teachers) to an understanding of the importance of critical evaluation, scrutinizing authorities, and the ability to generate knowledge through one’s own thinking. Finally, the *justification* dimension refers to the role of experiments and to how students evaluate claims. It ranges from denying the need for data and experiments to support arguments to the acceptance that knowledge is justified via a variety of thinking tools, experimentations, and observations (Conley et al., 2004).

In the present research, we follow this four-dimensional model of epistemic beliefs because these four dimensions of epistemic beliefs cover aspects that are regarded as important in learners’ development and in their role in learning (e.g., Mason, 2016). They have commonly been used in research on epistemic beliefs in science (e.g., Elby et al., 2016; Schiefer et al., 2017, 2020; Urhahne & Hopf, 2004). This dimensional structure is also in line with previous research (e.g., Hofer & Pintrich, 1997; Schommer, 1990). It has provided insights into individuals’ development, the effects of interventions, and relations with personal and academic variables in elementary school (Conley et al., 2004), middle school (J. A. Chen, 2012), and high school students (J. A. Chen, 2012; Kampa et al., 2016; Winberg et al., 2019). Some researchers have pointed out problematic aspects of normative descriptions of some epistemic beliefs (see e.g., Barzilai & Chinn, 2018; Hammer & Elby, 2002). We take the stance that for these four dimensions, the latter ends of their described continua (believing that scientific knowledge can change, that scientific answers can continuously develop, that it is important to critically reflect on sources of knowledge, and that evidence and arguments are the backbone of well-justified knowledge) represent desirable and sophisticated stances in the school context. Although these stances cannot be fully linked to an advanced evaluativistic level described by developmental models by Perry (1970) or Kuhn and Weinstock (2002), they are related to important aspects of evaluativism and represent rather sophisticated stances for the K-12 level.

Researchers have debated what stands behind the label “sophisticated” when referring to epistemic beliefs (e.g., Bråten et al., 2008; Elby & Hammer, 2001) and other similar terms, such as adaptive, availing, effective, high epistemic competence (e.g., Murphy & Alexander, 2016), constructivist positions (Muis et al., 2006), or apt epistemic performance (e.g., Barzilai & Chinn, 2018). In Conley et al.’s (2004) framework, a person who can be characterized as sophisticated does not strictly or always believe in external authorities, recognize the variability of answers in science, see knowledge as an evolving construct, or value evidence and experimentation in the process of acquiring new knowledge. In general, this interpretation is in line with more traditional approaches that have assumed that (nonreflected) absolute beliefs (i.e., a view of scientific knowledge as an accumulation of certain facts and absolute truths) obstruct learning and that multiplistic beliefs are beneficial (for a summary, see Rosman et al., 2017). The statements in the Conley et al.’s (2004) measure emphasize the relevance of evidence formation and critical thinking about (external) sources of knowledge, the importance of not blindly trusting experts, and the relevance of one’s own abilities and scientific thinking. These aspects are very relevant for reaching the (long-term) goals of students who take action and engage in 21s as responsible citizens in a society that is determined by science and technology (and are able to come to their own conclusions about questions such as vaccination, eating GMO food, and preventing climate change).

### Person-Centered Integrations of Dimensional and Developmental Models

More recently, the developmental and dimensional lines of research on epistemic beliefs have been integrated (although such integration was visible to a less systematic degree in earlier models, e.g., Bendixen & Rule, 2004; King & Kitchener, 1994). In the integrated view, the developmental stages that individuals typically go through are modeled across multiple dimensions of epistemic beliefs (Barzilai & Weinstock, 2015; Greene et al., 2008, 2010). In the present research, we follow this integrated perspective on epistemic beliefs.

Whereas researchers following the developmental perspective typically rely on qualitative assessments (Mason, 2016; Perry, 1970), researchers following the dimensional perspective usually implement quantitative assessments by employing Likert-scale questionnaires. Learners’ epistemic beliefs are then typically statistically modeled from a variable-centered perspective (e.g., DeBacker et al., 2008). Specifically, quantitative dimensions that are correlated with each other are modeled. Individuals have higher or lower values on each dimension, and these values represent a stronger or weaker affirmation of the respective epistemic belief. A common way of modeling epistemic beliefs in this way is by employing exploratory or confirmatory factor analysis (Clarebout et al., 2001; Conley et al., 2004; DeBacker et al., 2008; Merk et al., 2018; Winberg et al., 2019).

**Fig. 1:**
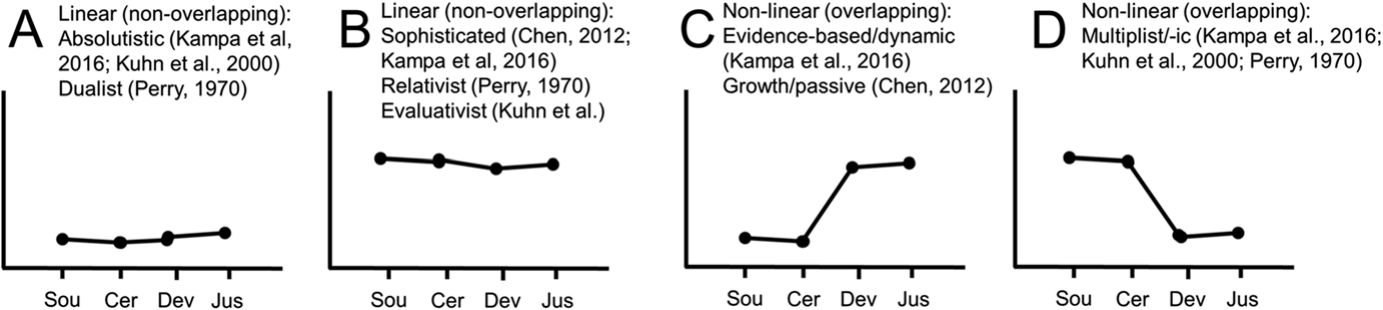
Developmental stages of epistemic beliefs across the four dimensions conceptualized by Conley et al. (2004) and references to their empirical sources. Note. Sou = source, Cer = certainty, Dev = development, Jus = justification of knowledge

In the integrated view of dimensional and developmental theories, a person-centered approach to the statistical modeling of epistemic beliefs has been introduced (J. A. Chen, 2012; Greene et al., 2010; Kampa et al., 2016). In the person-centered approach, epistemic beliefs assessed with Likert-scale questionnaires are not modeled as correlated dimensions. Instead, subgroups of individuals who show similar patterns of stronger or weaker epistemic beliefs across the different dimensions are modeled. This is usually achieved by conducting a latent profile analysis (Hickendorff et al., 2018). In a latent profile analysis, subgroups of individuals who show similar patterns of scores across different dimensions of epistemic beliefs are extracted in a data-driven manner. Examples of such patterns that have been theorized and found in empirical research are provided in Fig. 1. This approach can result in *linear*, *nonoverlapping* profiles of individuals who show a stronger or weaker affirmation of all epistemic belief dimensions (Fig. 1A and B). It can also reveal *nonlinear*, *overlapping* profiles of individuals who have stronger beliefs in some and weaker beliefs in other epistemic beliefs (Fig. 1C and D).

The combined approach of applying Likert-scale questionnaires and person-centered statistical modeling is congruent with the integrated conceptualization of epistemic beliefs as developmental stages across multiple dimensions (Greene et al., 2008, 2010). The assessment of different dimensions of epistemic beliefs is in accordance with the dimensional perspective, and the person-centered modeling approach across these dimensions enables the empirical examination of theorized developmental stages (Greene et al., 2008, 2010). In addition, particularly nonlinear profiles that can be discovered in person-centered statistical models would not be captured by variable-centered approaches (Edelsbrunner & Dablander, 2019; Hickendorff et al., 2018). Person-centered statistical modeling also has the advantage that it does not rely on arbitrary cut-offs (e.g., median splits) but instead extracts patterns empirically based on observed data for profile building. Thereby, the person-centered approach can provide more reliable and differentiated information about individuals’ epistemic beliefs across multiple dimensions (Kampa et al., 2016; Lonka et al., 2021).

However, the application of Likert-scale questionnaires to the assessment of epistemic beliefs has been criticized on theoretical and methodological grounds. Some arguments are that they exhibit unsatisfactory psychometric characteristics, that they are commonly worded in a domain-general rather than a domain-specific manner, and that it is not always clear which cognitive processes they evoke (for overviews, see Mason, 2016; Sandoval et al., 2016). Additionally, the assessment of epistemic beliefs with Likert-scale questionnaires might work best when instruments are adapted to specific age groups, particularly for younger participants (for a discussion on this, see Anschütz, 2012). Still, Likert-scale questionnaires remain the most common means for assessing epistemic beliefs and related constructs, particularly when collecting data from larger samples (for a recent review, see Lee et al., 2021; see also Rosman et al., 2017; Schiefer et al., 2020). In the present study, we contribute to the debate around Likert-scale questionnaires. We examine whether Likert-scale questionnaires produce comparable results regarding student profiles of epistemic beliefs and their relations with external student variables across multiple samples and studies when employing a person-centered statistical approach (i.e., latent profile analysis).

Recently, various studies on epistemic beliefs in the domain of science have been conducted from the person-centered perspective (J. A. Chen, 2012; Dai & Cromley, 2014; Ferguson & Bråten, 2013; Kampa et al., 2016; Trevors et al., 2017). An overview of profiles that have been found in these studies is provided in Fig. 1. For example, one profile is largely consistent with the developmental stages described as dualistic (Perry, 1970) or absolutistic (Kuhn & Weinstock, 2002; Kuhn et al., 2000). Mapped onto the four-dimensional conceptualization proposed by Conley et al. (2004), this profile describes students who exhibit strong beliefs that science knowledge is not created by oneself, is certain, does not develop over time, and is justified by authorities (Fig. 1A). A contrasting profile is presented in Fig. 1B, empirically identified by Chen (2012) and Kampa et al. (2016), and largely consistent with the developmental stages labeled reflective (King & Kitchener, 1994), relativistic (Perry, 1970), or evaluativistic (Kuhn et al., 2000). Additional profiles that showed differences in both level and shape have been identified (Figs. 1C and D; J. A. Chen, 2012; Kampa et al., 2016). In the present research, we integrate evidence from multiple studies to examine to which extent such profiles of epistemic beliefs in science are consistent across studies and show robust correlations with student variables.

### Relations Between Epistemic Beliefs in Science and Student Learning Characteristics

Previous research has investigated the relations between epistemic beliefs in science and other constructs that are related to students’ learning (e.g., motivation, cognitive abilities, learning strategies, learning outcomes; e.g., Mason & Bromme, 2010; Trautwein & Lüdtke, 2007; Tsai et al., 2011). These studies have mainly investigated relations between epistemic beliefs and science achievement (e.g., Greene et al., 2018) or motivational features (e.g., Buehl & Alexander, 2005; Mason et al., 2013). Results indicate that higher values on dimensions of epistemic beliefs typically seen as rather sophisticated are positively correlated with academic achievement (Greene et al., 2018) and the understanding of science (Elby et al., 2016). Results also indicate that more sophisticated epistemic beliefs go along with higher self-concept and self-efficacy (e.g., Buehl & Alexander, 2005; J. A. Chen, 2012; Mason et al., 2013). However, studies on the relations between epistemic beliefs and socioeconomic status (SES) have been inconsistent. Whereas some studies found a positive relation between more sophisticated epistemic beliefs and higher SES (e.g., Conley et al., 2004; Kampa et al., 2016; Ozkal et al., 2010), others could not replicate this relation (e.g., Trautwein & Lüdtke, 2007). Less research has focused on the relations between epistemic beliefs and specific science-related abilities, such as understanding of the control-of-variables strategy (CVS; Z. Chen & Klahr, 1999), an aspect that we will shed some initial light on.

Considering profiles of epistemic beliefs across multiple dimensions, only a few studies have examined how they are related to student characteristics (J. A. Chen, 2012; Greene et al., 2010; Kampa et al., 2016; Trevors et al., 2017; Urhahne, 2006). These studies have indicated that students with more sophisticated profiles show higher science achievement and grades, science achievement goals, and self-concept compared with other profiles (J. A. Chen, 2012; Mason et al., 2013; Urhahne, 2006). Compared with students in other profiles, students with less sophisticated profiles (e.g., uncommitted, absolutistic, or multiplistic) show lower motivation values and achievement scores and are associated with a lower social background. They also tend to be more present in nonacademic track schools than in academic track schools (J. A. Chen, 2012; Kampa et al., 2016). The variables investigated in such studies were sometimes assessed quite specifically (e.g., Trevors et al., 2017, investigated relations between profiles and the understanding of refutational texts), and studies have not been systematically replicated. These selective prior results limit the generalizability of conclusions about the relations of profiles of epistemic beliefs with covariates. In sum, systematic relations of epistemic belief profiles comprising a variety of covariates related to students’ learning have not been systematically investigated so far. In our study, we will provide a systematization by examining the relations of profiles of epistemic beliefs in science across the data from multiple studies.

### The Present Study: The Robustness of Profiles of Epistemic Beliefs and Their Relations to Student Characteristics

In the present study, we examined (a) whether and to what extent similar profiles of epistemic beliefs could be found across different studies and samples and (b) the degree to which profiles classified with the same label according to the integrated developmental-dimensional perspective show comparable patterns of relations with student variables across studies. To this end, we combined the original data sets from six studies that employed German adaptations of Conley et al.’s (2004) questionnaire for assessing four dimensions of epistemic beliefs in science. We decided to rely on German adaptations because various studies have employed Urhahne and Hopf’s (2004) German adaptation of Conley et al.’s (2004) questionnaire. In addition, limiting our study to the German context precludes confounding variables that could arise from including studies conducted in very different educational contexts and with adaptations of the instrument in different languages.

We collected six data sets that included 12 samples of students from different grades and tracks. In each of these samples individually, we employed a latent profile analysis. Across these individual analyses, we labeled the resulting profiles of epistemic beliefs in accordance with predefined criteria based on the existing literature on developmental stages and integrated models. Finally, we related the profiles to different student characteristics that were assessed in the respective studies. These characteristics included students’ grade and track, which provided insights into relations of students’ profiles with their school environment, as well as individual student characteristics (academic achievement, cognitive abilities, motivation, and SES). These steps allowed us to examine the extent to which profiles of epistemic beliefs in science that stem from studies from different areas, school types, and grades in Germany are comparable, in line with theories and prior findings about developmental stages, and show stable relations with external student variables across study contexts.

## Method

### Data Sets

We conducted a systematic literature search (using Scopus & Google scholar) in order to identify and gather all data sets that employed a variation of the Conley et al.’s (2004) questionnaire for the assessment of epistemic beliefs in the domain of science in the German language. Figure S1 documents the search process. We excluded 26 studies because they did not apply the Conley et al.’s (2004) instrument, did not assess all four dimensions of epistemic beliefs, or were not applied in German-speaking countries. We contacted the authors of the remaining eight studies to get access to the full data sets. Four of them could be included in our synthesis, supplemented by two—at that time point—unpublished data sets by the authors of the current review.

The resulting six data sets that were included in the present study comprised *N* = 10,932 students from elementary and secondary schools in Germany (ranging from Grades 3 to 12). In studies that covered multiple grades, we split the data sets into smaller sets that covered ranges of grades that correspond with the German school system. We identified Grades 3 and 4 as elementary school (which range from Grades 1 to 4 in the relevant federal states of Germany), Grades 5 and 6 as lower secondary school, Grades 7 to 9 as middle secondary school, and Grades 10 to 12 as upper secondary school. Splitting up the studies by these grade ranges resulted in 12 samples that stemmed from the six different studies (see detailed overview of the included studies and subsamples in the online supplemental material section S1 and Table S1). In addition to the labeling of grade levels as lower, middle, and upper secondary school, two types of tracks can be differentiated in German secondary schools. The academic track (*Gymnasium*) enables students to graduate with an A-level that permits university entry. The nonacademic track (*Realschule* or *Gemeinschaftsschule*) leads to a qualification that is equivalent to high school graduation and does not allow students to continue on to university studies.

The respective Ethics Committee for Psychological Research at the University or the respective Ministry of Education approved all studies. Prior to testing in all studies, parents’ gave written consent for their child’s participation.

### Measures

#### Epistemic Beliefs in Science

Epistemic beliefs were assessed by administering the 26-item instrument by Conley et al., (2004; German version by Urhahne & Hopf, 2004). The six studies included in this integrative study used slightly adapted versions of the translated questionnaire. The adaptations from the original instrument concerned, for instance, the item selection or the wording of the items. Four subscales reflected the dimensions of source (5 items, e.g., “Only scientists can observe natural phenomena”), certainty (6 items, e.g., “Scientific knowledge is always true”), development (6 items, e.g., “Sometimes scientists change their minds about what is true in science”), and justification of knowledge (9 items, e.g., “It is good to try experiments more than once to make sure of your findings”). We used within-sample *z*-standardized scores on these subscales to describe and compare the different profiles. In all studies, we recoded the source and certainty scales so that for each scale, higher scores reflected a stronger endorsement of a specific epistemic belief. As the epistemic beliefs questionnaire by Conley et al. (2004) was originally developed for students in Grade 5, think-aloud techniques were used in Study 1 prior to data collection to ensure that the 8- to 10-year-old children understood the wording and the meaning of the statements. Furthermore, the items were read aloud to the students to ensure that reading skills did not affect students’ understanding of the statements.

In addition to epistemic beliefs in science, the following covariates from our data sets were used to further investigate external relations of profiles of students’ epistemic beliefs. The covariates were selected because they represent central cognitive as well as non-cognitive variables that were used in prior research to some extent but also extended prior research (e.g., by including the control-of-variables strategy). We used only covariates that were assessed in at least two studies.

#### Socioeconomic Background (SES)

As an indicator of SES, the number of books at home (OECD, 2013) was assessed in a parent questionnaire in Studies 1, 4, and 6, and the highest International Socio-Economic Index of Occupational Status in the family (HISEI) was used in Study 5 (Ganzeboom et al., 1992). This index is based on the ISCO-88 (International Standard Classification of Occupations, see Wolf, 1997) as well as on further specifications of parents’ occupations and education levels.

#### Science Self-Concept

In Studies 1 (Sample 2), 4, 5, and 6, self-concept in the domain of science (for secondary school students in the domain of chemistry; for elementary school students in the general domain of experimentation) was assessed using 4-point Likert scales (e.g., “I’m good at doing experiments”). Internal consistencies were α_Study1_ = 0.88, α_Study4_ = 0.80, α_Study5_ = 0.94, and α_Study6_ = 0.90.

#### Cognitive Abilities

The studies used different measures of general cognitive abilities. In Study 1, fluid intelligence was measured with the *BEFKI-fluid* test (Schroeders et al., 2016). Within a time limit of 15 min, the children had to complete 16 series of figural patterns. In each case, they had to select the two subsequent figures. We used sum scores for our analyses (α = 0.85). In Studies 4 and 6, the General Cognitive Ability Test for Grades 4 to 12 (KFT; Heller & Perleth, 2000) was used to assess cognitive abilities.

#### Control-of-Variables Strategy

The CVS is a central aspect of scientific reasoning and reflects students’ understanding that in a valid experiment, only one variable at a time can be varied, which is an important prerequisite for the design of unconfounded experiments (see Z. Chen & Klahr, 1999; Zimmerman, 2007). The CVS was operationalized in different ways in Studies 1 and 6 for the different age groups. In Study 1, students’ understanding of the CVS was assessed with six single-choice items with three answer alternatives (one correct, two misconceptions), which were developed for the target group of elementary school students (see Koerber et al., 2015; Mayer et al., 2014). The items were presented in everyday-life contexts, illustrated with pictures, and designed to apply the CVS in the context of domain-general experimentation tasks (an example can be found on p. 160 in Schiefer et al., 2019). The items were scored dichotomously (1 = *correct answer*, 0 = *wrong answer*). In Study 6, the measure was composed of five Likert-scale questions (e.g., “When scientists conduct experiments to find out whether a variable influences a characteristic, then they vary all things at the same time that could have an influence”), with four answer options ranging from 1 (*do not agree at all*) to 4 (*fully agree*). Internal consistencies were α_Study1_ = 0.66 and α_Study6_ = 0.72.

### Analytical Approach: Modeling and Interpreting Student Profiles of Epistemic Beliefs

We modeled the data from each of the 12 samples separately. Integrating and analyzing the different data sets within the same analysis (Curran & Hussong, 2009) was not appropriate with our data sets because they were based on different adaptations of the Conley et al. (2004) questionnaire with regard to number, type, and order of items, preventing linking and measurement invariance analysis across studies. We first computed descriptive statistics and estimated internal consistencies separately for each sample. Then, we conducted the main analysis—a latent profile analysis—again separately for each sample, to identify student profiles of epistemic beliefs across the four indicator variables in each sample. The four indicator variables contained each student’s *z*-standardized mean scores on beliefs in certainty, development, source, and justification of knowledge, respectively.

A central decision regarding the analytical approach was to *z*-standardize students’ four scores representing their epistemic beliefs within each of the 12 samples before extracting and interpreting the epistemic belief profiles. We decided to base the profile labels and interpretations predominantly on within-sample *z*-standardized estimates because different Likert-scale formats were used in the different studies, and these differences could be reconciled by placing the results from all the studies on the common *z*-scale. Furthermore, the labels of the Likert-scale options might not reflect the exact level of epistemic belief that was indicated by the respective label, which has been shown with detailed interview methods (Krettenauer, 2005). In addition, prior studies and theoretical considerations have indicated that students’ epistemic beliefs are affected by the epistemic climate in the classroom and the broader school environment (e.g., Muis & Duffy, 2013), supporting the decision to relate their strength of beliefs to that of their respective sample. For these reasons, we *z*-standardized individual students’ mean scores on each dimension of epistemic beliefs by using their respective sample’s mean and variance in order to undertake the latent profile analyses. We did however ensure that the meaning of the resulting profiles of epistemic beliefs was also based on the total sample across the 12 included studies by defining the profile criteria that are described next.

Prior to the latent profile analyses, we defined a priori criteria for profile classifications. This means that for each profile of epistemic beliefs found in any of the samples, we provided a label (e.g., *absolutistic* or *multiplistic*) that described the respective profile. In individual empirical studies employing latent profile analyses, profile labels are usually defined after the profiles have been extracted and are based on theoretical considerations. In the present case, we defined criteria that could be applied equally well to any profile that resulted from any data set. By doing so, we could compare and contrast the meaning of the resulting profiles on the basis of the same criteria.

We defined the criteria for labeling the profiles on the basis of careful considerations that were derived from consulting theoretical and empirical research on the developmental stages of epistemic beliefs and their integration with dimensional models (J. A. Chen, 2012; L. E. Ferguson & Bråten, 2013; Greene et al., 2018; Kampa et al., 2016; King & Kitchener, 1994; Kuhn et al., 2000; Perry, 1970; Trevors et al., 2017; Urhahne & Hopf, 2004). The basis for the profile criteria were the four kinds of profiles that were outlined and empirically supported in prior studies (see Fig. 1): absolutistic, multiplistic, evidence-based, and sophisticated. On the basis of the theoretical descriptions and prior empirical findings on these profiles, we derived a total of seven labels for the profiles in our integrative study. The seven kinds of profiles are outlined in Table 1. On the basis of the prior literature, we had to define in detail which patterns in estimates of students’ *z*-standardized mean scores across the four dimensions of epistemic beliefs would receive which label. To this end, we defined the criteria that are presented in Table 1. We took into account three kinds of criteria for labeling and describing the profiles of epistemic beliefs. These included (a) sample-general criteria based on the level of the strength of students’ epistemic beliefs (see the *level criteria regarding the total data set* in Table 1), (b) sample-specific criteria based on the level of the strength of students’ epistemic beliefs relative to their respective sample (see the *level criteria regarding the individual samples* in Table 1), and (c) shape criteria used to define the profiles according to the relative level of the strength of the four beliefs compared with each other (see the *shape criteria* in Table 1). The (a) *level criterion regarding the total sample* describes how many dimensions in a profile are situated below or above the overall mean of the total sample of students from all studies (not weighted by sample size). We included this criterion in order to prevent profiles from receiving a certain label (e.g., multiplistic) exclusively on the basis of the z-standardized values within each sample. For example, in order to receive the label multiplistic, a profile had to exhibit mean scores that were above the mean of the total sample of students from all studies on the source and certainty dimensions along with means that were below the total sample mean on the development and justification dimensions. This first criterion ensured that in comparison with the total sample of students from elementary school to the end of secondary school, profile labels reflected stronger and weaker beliefs, respectively, than what the average was during this entire developmental period according to our data. The (b) *level criterion regarding the individual sample* depicts whether a profile was situated below, close to, or above the mean of the specific sample out of the total of 12 samples that were analyzed. For example, in order to receive the label multiplistic, a profile had to exhibit mean scores that were above the mean of the specific sample of students on the source and certainty dimensions as well as means that were below the specific sample mean on the development and justification dimensions. Finally, the (c) *shape criterion* defines the extent to which dimensions within a profile differed across the four dimensions. This criterion was also based on the *z*-standardized scale within each sample. We used standard deviations to define limits for labeling profiles as being overlapping or nonoverlapping (see Table 1 & also Fig. 1). This criterion ensures that the profile labels validly represent theoretical assumptions about profiles such as that the multiplistic profile showed substantially higher means on the source and certainty dimensions than on the development and justification dimensions.

**Table 1:** Criteria applied for identifying profiles within a given sample

Profile	Level criterion regarding the total sample	Level criterion regarding the individual samples	Shape criterion	Description
Highly sophisticated	Majority of dimensions 1 standard deviation (SD) above the total sample mean (TSM)	Majority of dimensions 1 SD above the individual sample mean (ISM)	Nonoverlapping profile, the difference between the dimensions should be less than 1 *SD*	Students in this profile show very well-developed epistemic beliefs on most of the dimensions in a consistent manner. Typically, students belonging to this profile fall at the upper ends of the Likert scales
Sophisticated	Majority of dimensions above the TSM but not more than 1 SD	Majority of dimensions above the ISM but not more than 1 SD	Nonoverlapping profile, the difference between the dimensions should be less than 1 SD	Students in this profile show well-developed epistemic beliefs on most of the dimensions in a consistent manner. Compared with other students in the same sample and in the total sample, they do not outperform other students in such a strong manner that this would justify an interpretation as being a highly sophisticated profile. Often, these profiles do not reach the maximum value on the Likert scale
Absolutistic	Majority of dimensions below the TSM	Majority of dimensions below the ISM	Nonoverlapping profile, the difference between the dimensions should be less than 1 SD	This profile comprises students who score comparatively low on the Likert scales. Although they might score in a medium range on the raw data, they show more disagreement with adequate epistemic belief statements than the students around them and the total sample
Intermediate	Two dimensions (any) above the TSM, two dimensions below the TSM but not more than 1 SD	Two dimensions above the ISM, two dimensions below the ISM but not more than 1 SD	Nonoverlapping profile, the difference between all dimensions is less than 1 SD	The intermediate profile comprises students who score close to the sample means on all dimensions. This points to a low differentiated profile that resembles the *uncommitted* profile found by Chen (2012). These students are rather reluctant to take a particular epistemic position
Evidence-based	Dev and Jus dimensions above the TSM, Sou, and Cer dimensions below the TSM	Dev and Jus dimensions above the ISM, Sou, and Cer dimensions below the ISM	Overlapping profile, the difference between the dimensions (Dev/Jus vs. Sou/Cer) should be more than 1 SD	Based on work by Kampa et al. (2016), this overlapping profile describes students who emphasize the importance of experimentation and the role of empirical data in science (the importance of evidence), which is one aspect of evaluativism (Kuhn et al., 2000). To a comparatively strong degree, these students accept that scientific knowledge evolves over time. Only external authorities are recognized as sources of scientific knowledge. Scientific knowledge does not change, and scientific questions have only one answer
Multiplistic	Sou and Cer dimensions above the TSM, Dev, and Jus dimensions below the TSM	Sou and Cer dimensions above the ISM, Dev, and Jus dimensions below the ISM	Overlapping profile, the difference between the dimensions (Sou/Cer vs. Dev/Jus) should be more than 1 SD	The multiplistic profile arose from work by Perry (1970) and incorporates an important developmental perspective as it might constitute a transition stage between rather informed and rather naïve epistemic beliefs. Students in this profile agree that science does not necessarily need to be produced by external authorities; many people can make comments or contribute to generating knowledge. The importance of supporting scientific knowledge with evidence is comparatively underestimated so that the idea that knowledge changes over time is rather disregarded
Mixed	Mean values of students in this profile do not meet the specified criteria for any a priori determined profiles			

Note: *TSM* total sample mean, *ISM* individual sample mean, *Sou* source, *Cer* certainty, *Dev* development, *Jus* justification of knowledge

Using these criteria, we were able to apply transparent and objective rules for interpreting and comparing profiles within and across all samples. In this way, discrepancies due to subjectivity within interpretations were minimized. We decided not to apply significance tests (e.g., to test whether a profile mean was significantly above a sample mean) because the profiles were expected to differ greatly in sample sizes, which would imply drastically different statistical power and thus unknown reliability for inferential statistical tests across profiles and samples. Thus, in this rather exploratory (although theory-driven) context, we decided to rely on descriptive interpretations of model estimates. Details of the analytic approach for estimating the latent profiles within each sample are described in the online supplementary materials under section S2.

## Results

We first report the descriptive statistics for all the samples, followed by the results on the student profiles that were extracted for epistemic beliefs in each sample. Finally, we present relations of students’ grade and track with the prevalence of the epistemic belief profiles as well as relations with students’ individual characteristics.

### Descriptive Statistics

Table S2 in the online supplementary materials presents the means and standard deviations of the epistemic belief dimensions, the number of items per dimension, and Cronbach’s alphas for each of the 12 samples. The employed Likert scales (based on the labeled rating scales that ranged from 1 to 4, 1 to 5, or 1 to 6 in the different studies) should be considered when interpreting the descriptive statistics within the respective samples. The internal consistencies (Cronbach’s Alpha) ranged from 0.59 to 0.82 for the source dimension, from 0.57 to 0.81 for certainty, from 0.57 to 0.86 for development, and from 0.60 to 0.81 for justification.

### Profiles of Epistemic Beliefs Within and Across Samples

**Fig. 2:**
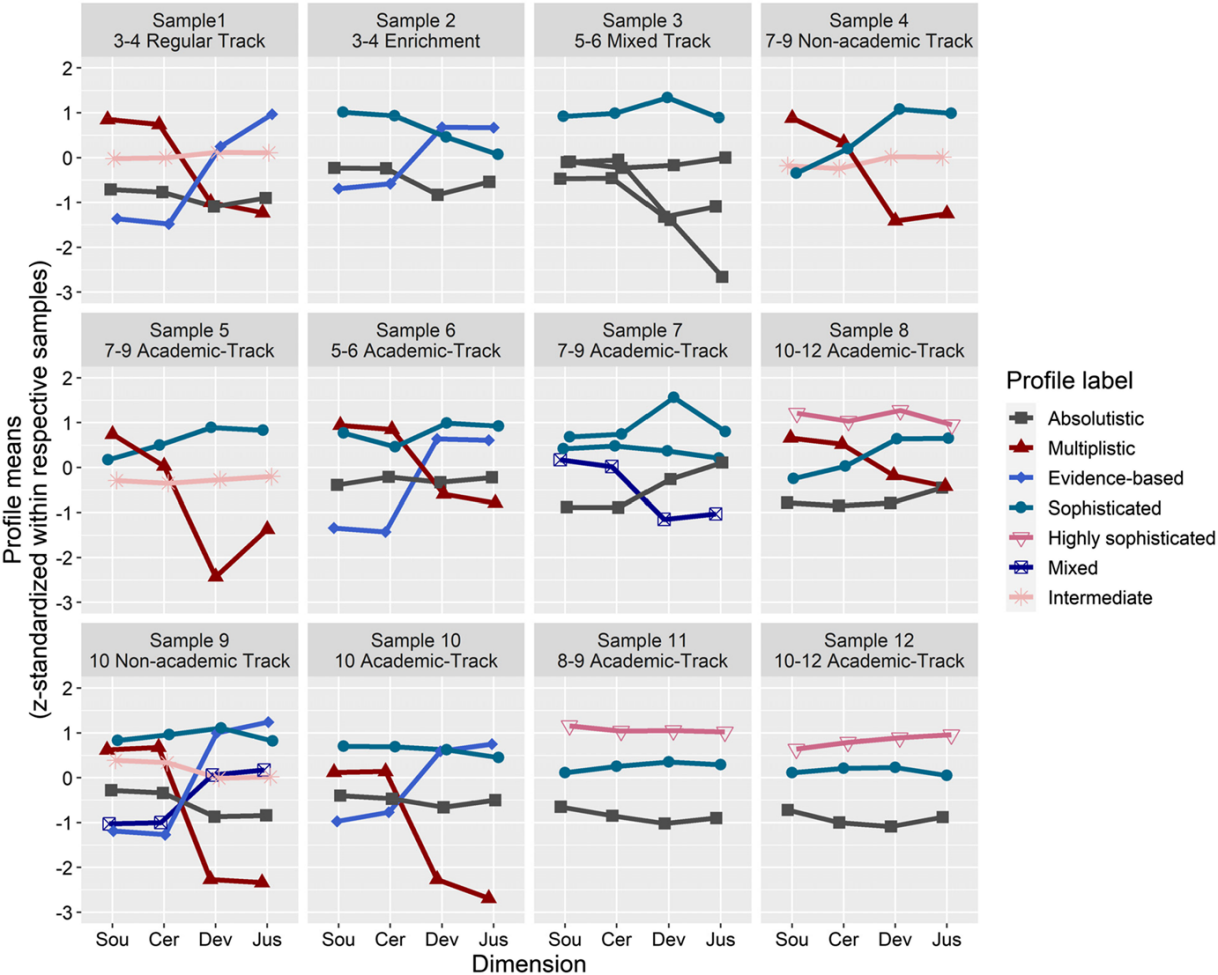
Profiles of epistemic beliefs in each sample. Note. Sou = source, Cer = certainty, Dev = development, Jus = justification

We applied the criteria that were described for model selection (see Table 1) in each of the 12 samples (see model fit indices for the latent profile analyses with one to six profiles in each sample in Table S3 in the online supplemental materials). The student profiles of epistemic beliefs identified by following the previously defined criteria (see Table 1) and their frequencies within the 12 different samples are depicted in Fig. 2 and Table 2, respectively.

**Table 2:** Percentages of students in each profile per sample

Profile	Sample											
	EL 3-4I	EL 3-4II	MS 5–6	NAS 7–9	AS 7-9I	AS 5–6	AS 7-9II	AS 10-12I	NAS 10	AS 10	AS 8–9	AS 10-12II
Highly sophisticated	-	-	-	-	-	-	9	9	-	-	12	24
Sophisticated	-	30	19	26	35	21	41	29	19	42	56	47
Absolutistic	30	39	71	-	-	50	30	31	19	-	32	29
Intermediate	44	-	-	53	52	-	-	-	32	40	-	-
Evidence-based	10	31	-	-	-	10	-	-	8	14	-	-
Multiplistic	16	-	-	21	13	18	-	31	5	4	-	-
Mixed		-	-	-	-	-	19	-	16	-	-	-

*Note*.—= Profile does not occur in this sample. See Table S1 for the sample abbreviations

We provide an example to clarify the labeling of the profiles according to the labeling criteria a to c. For instance, in Profile 1 in Sample 1 (triangle and red in Fig. 1), (a) the means of the source (*M* = 0.85) and certainty (*M* = 0.74) dimensions were above the total sample means (source: *M* = 0.46, certainty: *M* = 0.62), and the means of the development (*M* = -1.00) and justification (*M* = -1.23) dimensions were below the total sample means (development: *M* = -0.21, justification: *M* = -0.68). Furthermore, (b) the source (*M* = 0.85) and certainty (*M* = 0.74) dimensions fell above the individual sample mean (*M* = 0.00), and the development (*M* = -1.00) and justification (*M* = -1.23) dimensions fell below the individual sample mean. Finally, the shape criterion (c) pointed to a clearly overlapping profile, as the difference between the source/certainty dimensions compared with development/justification was more than one standard deviation (Diff _S/C – D/J_ = 1.91). Thus, we labeled this profile *multiplistic*.

More than one profile received the same label within a sample when several profiles met the defined criteria (e.g., we identified three absolutistic profiles in Sample 3). We found at least one *absolutistic* profile in all samples except Samples 4 and 5. A *multiplistic* profile was found in seven of the 12 samples, and an *evidence-based* profile was found in five of the 12 samples. Students in the evidence-based profile (which was named to correspond to a similar profile found by Kampa et al., 2016) are characterized by an emphasis on evidence that is reflected by high scores on the development and justification dimensions. At least one *sophisticated* profile appeared in all of the samples except Sample 1 (elementary school, regular track), and a *highly sophisticated* profile appeared only in Samples 8, 11, and 12 (all academic track samples). A *mixed* profile (which met none of the defined criteria) was found only in Samples 7 and 9, and an *intermediate* profile was found in Samples 1, 4, 5, and 9. To provide further information, we generated an interactive app (https://peter1328.shinyapps.io/EpistemicBeliefsinScience_ProfileApp/) that enables readers to visualize the respective profiles in all individual samples on different scales (e.g., original Likert scale vs. *z*-standardized scale) and with different comparison variables (e.g., means & standard deviations of the total sample).

### Relations of Profiles with Grade and Track

**Fig. 3:**
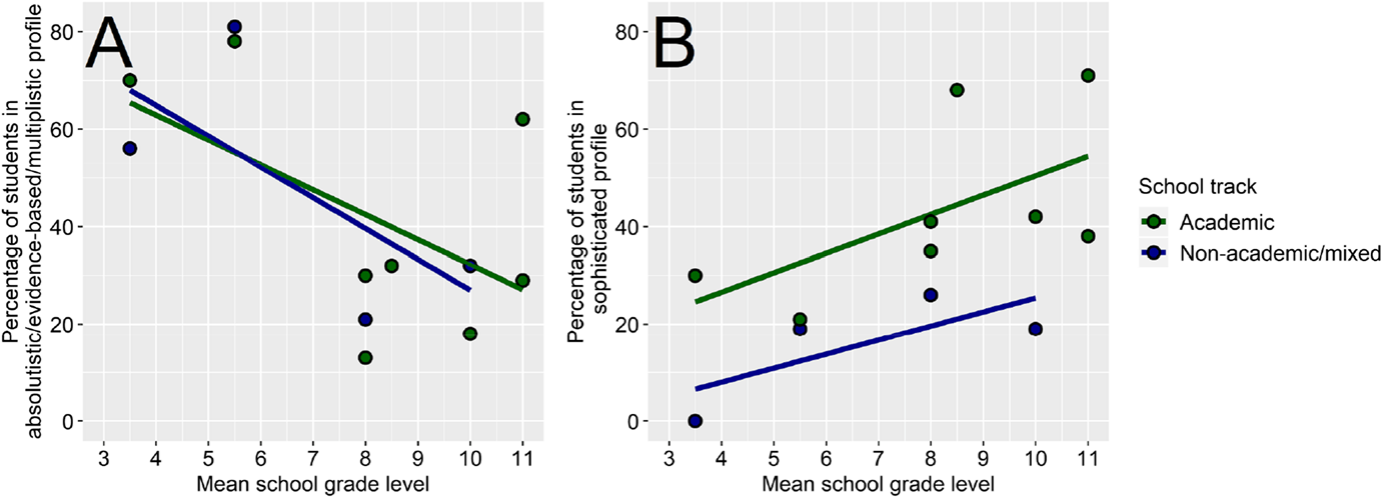
Relations between epistemic belief profile types A and B and grade level per track (academic vs. nonacademic). Note. A = *y*-axis depicts the percentage of students in an absolutistic/evidence-based/multiplistic profile. B = *y*-axis depicts the percentage of students in a sophisticated profile. Dots: percentages in each sample; lines, linear regression lines across mean grade for the two track groups

In order to relate the occurrence of the different profiles to students’ grade and track, we grouped the profiles into two groups. First, the group of less sophisticated profiles, including those profiles which fully (absolutistic) or partially (evidence-based, multiplistic), exhibited low means on at least two dimensions of epistemic beliefs. Second, those profiles in which all means were relatively high and could thus be described as corresponding to the highest developmental level (sophisticated, highly sophisticated; e.g., Kuhn et al., 2000). We related the percentages of students in these two different types of profiles to their grade and track (see Fig. 3). The descriptive results (inferential statistics would not be informative with this number of data points) indicated that across both the academic and nonacademic/mixed tracks, samples that had younger students contained more students who had absolutistic, evidence-based, or multiplistic profiles. More students with a sophisticated profile were found in academic track schools compared with nonacademic track schools and in higher compared with lower grade levels.

### Relations Between Profiles and Student Characteristics

**Fig. 4:**
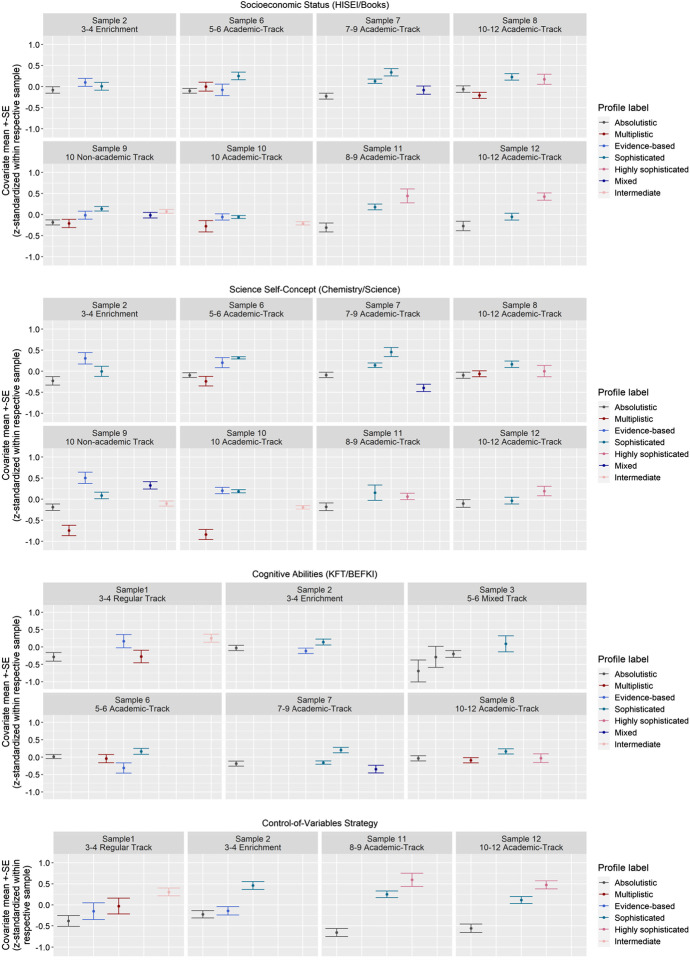
Z-standardized covariate means and standard errors of the different profiles in each sample

Figure 4 presents the relations of epistemic belief profiles with the student covariates SES, science self-concept, cognitive abilities, and the CVS.

SES (assessed in eight samples) tended to be lower in the absolutistic and multiplistic profiles and higher in the sophisticated and highly sophisticated profiles. The evidence-based profile was found in four out of the eight samples in which SES was assessed. In three out of these four samples, the evidence-based profile was related to higher levels of SES than the absolutistic and multiplistic profiles, but to lower levels of SES than the sophisticated and highly sophisticated profiles. The mixed profile (identified in two out of the eight samples) showed about average levels of SES, and the intermediate profile (identified in one out of the eight samples) showed slightly above-average SES. Furthermore, the overall range of *z*-scores of SES across profiles ranged from -0.25 to 0.45, indicating a rather substantial discriminating value of the profiles regarding SES. Overall, our results point to a positive relation between the level of sophistication of epistemic belief profiles and SES.

Science self-concept was assessed in eight samples. The absolutistic profile (identified in seven out of the eight samples) and the multiplistic profile (identified in four out of the eight samples) always showed the lowest and below-average levels of science self-concept. The evidence-based profile (identified in four out of eight samples) showed above-average and the highest means in science self-concept out of all the profiles. The sophisticated profile (identified in all eight samples) and the highly sophisticated profile (identified in three out of the eight samples) showed average science self-concept values in a few samples and above-average levels in the remaining samples. Overall, this comparison points to a diverging pattern regarding the two nonlinear profiles: The multiplistic profiles showed similar and sometimes even lower levels than the linear absolutistic profiles; by contrast, the evidence-based profiles showed similarly high and even higher levels than the linear sophisticated profiles. The mixed profiles showed an above-average level once and a below-average mean estimate once. The intermediate profile showed an average estimate. Overall, there was a tendency in our results for students with a high self-concept to be assigned to (highly) sophisticated or evidence-based profiles. However, this result was not consistent across samples.

Cognitive abilities were assessed in six samples. We found eight absolutistic profiles in these samples; all of them showed average or slightly below-average cognitive abilities. Students in the multiplistic profile (three out of the six samples) also exhibited average or slightly below average cognitive abilities, whereas students in the evidence-based profile showed below-average cognitive abilities in two samples and slightly above-average cognitive abilities in one sample. The sophisticated profile (identified six times in five samples) as well as one highly sophisticated profile showed an inconsistent pattern (once slightly below average, twice about average, and four times above average). We also found one mixed profile that showed below-average cognitive abilities and one intermediate profile that showed above-average cognitive abilities. Even though these results were not consistent across profiles, they point to above-average cognitive abilities in (highly) sophisticated profiles.

The CVS was assessed in four samples. The absolutistic profile (identified in all four of these samples) always showed below-average and the evidence-based profile (identified in two out of the four samples) slightly below-average estimates. The sophisticated profile (identified in three out of the four samples) consistently showed above-average means. The highly sophisticated and the intermediate profiles clearly showed above-average estimates in three samples. The multiplistic profile (identified in one out of the four samples) showed an average estimate. Overall, these results point to a positive relation between the level of sophistication of the epistemic belief profiles and the understanding of the CVS.

## Discussion

This study presents an integration of six data sets encompassing 12 samples that examined profiles of epistemic beliefs in elementary and secondary school students in Grades 3 to 12. We applied a latent profile analysis in each sample and found that most student profiles of epistemic beliefs were in accordance with prior theories and findings and could be replicated across multiple samples or studies. In addition, we found indications that the frequency of different profiles varied across grades and tracks and the profiles showed various systematic mean patterns on covariates in multiple samples. We discuss these results and their implications in turn.

### The Comparability of Epistemic Belief Profiles Across Multiple Samples

The first finding from our study is that similar profiles of epistemic beliefs in science (i.e., absolutistic, multiplistic, evidence-based, sophisticated) could be found across different samples. The profiles were identified despite the use of slightly different versions of the Conley et al.’s (2004) questionnaire in the different studies, which points to the robustness of profiles regardless of which specific item versions are used. The majority of these profiles can be ascribed to labels of developmental stages described in prior literature (J. A. Chen, 2012; Kuhn et al., 2000; Perry, 1970). We found all four profiles discussed in prior research (see Fig. 1) in multiple samples of different grades and tracks. To the best of our knowledge, this is the first systematic evidence that points to a moderate to strong robustness and comparability of profiles of epistemic beliefs in science across different samples, age groups, and tracks.

We found the two overlapping profiles (multiplistic & evidence-based) across multiple samples and also found both profiles in the same samples. In addition, in some samples, rather high percentages of students showed one of these profiles, the highest percentage being 31% in an elementary school sample. These findings indicate that these two types of profiles do not present a phenomenon of a developmental stage that occurs in just a few students or in very specific contexts. Instead, developmental stages in which epistemic beliefs are strong in some dimensions—in a direction that is commonly described as more advanced (e.g., Kuhn et al., 2000)—but rather weak in others are a systematic phenomenon across grades and tracks. In addition, even though all samples came from Germany, the federal states of Germany have highly independent and different educational systems. Therefore, we showed that these profiles appear across a variety of educational contexts.

In addition to profiles described in prior literature, we also found two types of profiles that had not been given systematic labels before. We labeled the first type *intermediate.* This new profile described students with about-average means on all four dimensions of epistemic beliefs. We named the second type *mixed* for two profiles that exhibited moderately low levels on the same dimensions as the multiplistic and evidence-based profiles, respectively, and had means close to the sample average on the two remaining dimensions. A profile resembling the intermediate profile also emerged in the study by Chen (2012). Chen (2012) labeled this profile of students who were fairly hesitant to commit to a particular position of beliefs *uncommitted*. In our study, a substantial number of students across five samples was assigned to an intermediate profile (between 32 and 53%, see Table 2). These students could not be characterized by a specific position regarding the nature of knowledge and knowing in science, nor could they be characterized by a specific pattern in covariates. Most student variables showed average values. Thus, an adequate fostering and support of this relatively large group, which could also be labeled uncommitted (see J. A. Chen, 2012), passive, or even uninvolved, should be taken into account. The two mixed profiles strongly resembled the two overlapping (multiplistic & evidence-based) types of profiles, but they did not fulfill all of the a priori criteria that we had developed for labeling these profiles. We assume that the intermediate and mixed profiles represent intermediate steps that lead from one profile to the other. For example, the intermediate profile might represent students who are on the verge of developing a more refined structure of epistemic beliefs that will lead to a new profile (e.g., the sophisticated profile). Such intermediate steps might be related to the developmental steps found in earlier fine-grained models that were neglected in later models in favor of simplicity, such as those by Kitchener and King (1981) and Perry (1970). For example, King and Kitchener (1981) described a stage (*Stage 3* therein) in which individuals assume that absolute knowledge might sometimes exist but not always, and as long as there is not sufficient evidence for any specific claim, any claims that people make are justified. Perry (1970) described a stage (*Position 4* therein) in which it is acknowledged that authorities make evaluativistic judgments about knowledge, but this is viewed as an act on their side rather than part of the nature of knowledge. Such ambivalent opinions toward the acknowledgment of the importance of evidence, the certainty of knowledge, and the roles of authorities as well as the self might capture individuals in the intermediate profile. Whether this temporal assumption holds true should be examined in future longitudinal studies applying the person-centered approach.

### Relations of Epistemic Belief Profiles With Grade and Track

The comparisons between different grades and tracks enabled overarching observations and conclusions. The absolutistic and overlapping profiles seemed to be more prevalent in samples comprising students from lower grades and from nonacademic as well as mixed tracks. Profiles commonly labeled sophisticated showed a higher prevalence in samples comprising students from higher grades and from academic tracks. These results might be associated with the epistemic climate in class (see Feucht, 2010; Muis & Duffy, 2013), which can be assumed to be more advanced in academic track schools and in higher grades compared with nonacademic tracks and lower grades. For higher ages and tracks, more emphasis might be placed on constructivist teaching practices such as critical discussion and thinking, comparisons of different approaches, or finding one’s own solutions for scientific problems, all of which have been shown to positively affect changes in students’ epistemic beliefs (Bendixen & Feucht, 2010; Muis & Duffy, 2013). In addition, academic track and nonacademic track schools in Germany follow different curricula. Curricular differences between the two types of schools might result in differences in the quantities and degrees of abstraction. As a hypothesis, we deduce that richer and more abstract scientific content in the academic track might be more suitable for reflecting on the ways of thinking and working in the science disciplines. At the same time, even the simplest scientific models (e.g., the simple particle model) or experimental procedures (e.g., most prominently, the phenomenon-oriented experiments that involve a candle by Faraday & Crookes, 1861) can lend themselves to epistemic reflection. From the perspective of further research, we propose that future studies should examine the interplay between the selection of specific science content and how it is embedded in teaching–learning processes in shaping students’ epistemic beliefs. Overall, our results point to the postulated further development of epistemic beliefs with age and school education, as well as a decrease in more naive or heterogeneous views as postulated by developmental models (see Kuhn & Weinstock, 2002). These results indicate that science profiles may homogenize (i.e., the profiles might overlap less) in higher grades and academic tracks. However, as opposed to developmental models that postulate continuous progress from one level to another, our results point to two more differentiated processes. On the one hand, the transition from one developmental level to another might not represent linear progress. On the other hand, within a single age group, we found huge individual variability represented in multiple profiles per age group. This differentiation is supported by our observation that, for instance, sophisticated profiles also exist in samples with younger students, such as an elementary school sample, which includes 8- to 9-year-old children. From a theoretical point of view (see Kuhn & Weinstock, 2002), students at this age would rather have been expected to be on a realistic or absolutistic level. However, this observation goes hand in hand with prior studies that also found epistemic diversity, as well as sophisticated profiles in samples with younger students, such as in elementary school (e.g., Mansfield & Clinchy, 2002). Our finding complements recent research that has described individual differences in epistemic understanding and broader scientific thinking in students as young as elementary school (e.g., Mayer et al., 2014; Schiefer et al., 2021; Schlatter et al., 2021), how they develop from one profile of scientific reasoning into another (Schlatter et al., 2021), and how such development can be supported by targeted interventions (e.g., Schiefer et al., 2017, 2020). Our finding that absolutistic or evidence-based profiles were identified in students from middle or higher grades further contributes to these findings of substantial individual variability.

Future research addressing this epistemic diversity might benefit from approaches from the field of inclusive science education (Stinken-Rösner et al., 2020). Inclusive approaches are aimed at supporting all learners by exploiting the diversity of a group as a resource and an opportunity for individual and joint learning processes (Florian & Spratt, 2013). One such approach, Universal Design for Learning (CAST, 2018), has recently been employed for individualizing access to epistemic reflections in inclusive classrooms and for helping to foster epistemic beliefs in heterogeneous learning settings (Roski et al., 2021). Future studies might follow up on the idea of beneficial epistemic diversity by implementing similar collaborative settings in which students coming from different profiles work and reflect together.

### Relations of Profiles With Student Covariates and Implications for Science Education

We examined the degree to which profiles of epistemic beliefs showed comparable patterns of relations with external student variables (SES, science self-concept, cognitive abilities, & the CVS) across samples. Some of the student variables were not considered in previous research (e.g., the CVS); others have only been related to general epistemic beliefs (e.g., cognitive abilities; see Trautwein & Lüdtke, 2007). Prior research on the relations of profiles with SES and self-concept (e.g., J. A. Chen, 2012; Kampa et al., 2016) has lacked a systematic integration of evidence from multiple studies. We contributed to closing this gap by providing systematic evidence on relations to various external criteria that are relevant to science education across multiple studies.

Overall, relations with external covariates corresponded to normative expectations provided by developmental models of epistemic beliefs in science (e.g., Kuhn & Weinstock, 2002; Perry, 1970). We found desirable relations for more sophisticated profiles, undesirable relations for absolutistic profiles, and intermediate relations for the evidence-based and multiplistic profiles. However, we also revealed exceptions to these expected patterns (e.g., inconsistent relations with cognitive abilities & epistemic belief profiles).

Regarding student characteristics, the sophisticated profile is characterized by a rather high SES, an average to above-average science self-concept, mostly above-average cognitive abilities, as well as a strong understanding of the CVS, the latter being central for science understanding and valid scientific conclusions (Z. Chen & Klahr, 1999; Schwichow et al., 2020; Zimmerman, 2007). These relations indicate that students in this profile are advanced overall and have advantages in many criteria (e.g., SES & cognitive abilities). In particular, they demonstrate the strongest understanding of the CVS and therefore a strong understanding of the genesis of scientific evidence and theory. Students in the sophisticated profile have the potential to enrich society in scientific domains, for example, by generating new ideas and finding solutions for social, economic, or environmental problems. Thus, it might be beneficial to foster the science abilities of these students (e.g., by having them participate in early student science competitions) and motivate them to pursue STEM careers (e.g., Robertson et al., 2010). These findings also provide an alternative, empirical perspective on debates regarding what should be considered epistemic sophistication (Elby et al., 2016; Rosman et al., 2017). The finding that students in this profile, which has been ascribed rather high sophistication from a developmental perspective (Kuhn et al., 2000), showed the highest mean values on other desirable student characteristics might be interpreted as support for this profile’s labeling and interpretation.

The absolutistic profile, by contrast, is characterized by rather low SES, average self-concept, average to low cognitive abilities, and a weak understanding of the CVS. This pattern points to specific disadvantages and low abilities, which makes them a “group at risk” regarding participation. From a societal point of view, it appears particularly important to foster the motivation as well as the epistemic cognition of these students. Recent political decisions during the COVID-19 pandemic or debates about climate change require an advanced understanding of the particular characteristics of science and scientific knowledge. Epistemologically more advanced students might be less likely to be prone to misinterpretations of science in filter bubbles, echo chambers, fake news, alternative facts, and intentional disinformation. The comparatively large percentages of students in the absolutistic profile (between 19 and 50% per sample) stress the importance of fostering epistemic beliefs as an explicit learning goal in science lessons. Supporting those students’ epistemic beliefs might be achieved by providing a rich epistemic climate in the classroom (Muis & Duffy, 2013). Specifically, for students in the absolutistic profile, a combination of constructivist practices with rather strong teacher guidance might be beneficial (Greene & Yu, 2015). This combination has been shown to be able to facilitate the development of more advanced epistemic beliefs in learners starting in elementary school (Bendixen, 2016). Students in this profile appear to be generally among those learners with lower preconditions for learning. Teachers might facilitate the development of more advanced epistemic beliefs in these students by supporting them individually within the exploration of epistemic problems that challenge their prior epistemic beliefs (Lehrer et al., 2008). In order to be able to elevate students’ epistemic beliefs, such support should optimally encompass targeted, explicit reflection on the meaning of the respective inquiry activities for the construction of knowledge (Kittleson, 2011).

The multiplistic profile is also related to average to low SES, the lowest self-concept in science, as well as average cognitive abilities and an average level of understanding of the CVS. Students in these profiles seem to be critical of authorities and the credibility of science. Their motivation might be fostered by providing clear and accurate feedback on their ability and self-efficacy as well as classroom discourse that focuses on the importance and utility of science content and ability. As they also seem to be ignorant or inexperienced with respect to the importance of evidence or experiments, two measures could enhance their epistemic beliefs in science. First, they could benefit from being offered interesting and engaging activities (see Linnenbrink-Garcia et al., 2016; Pintrich, 2003), and second, from learning about the scientific method and inquiry-based learning (e.g., Brickman et al., 2009; Furtak et al., 2012). Moreover, stressing the trustworthiness of scientific knowledge due to rigorous empirical testing might be the most important learning step for enabling students in this profile to move to a more advanced level. It might be helpful for educators to carefully introduce the constructivistic nature of epistemology in science and to be aware of the *perils of epistemic relativism* (Romero-Maltrana et al., 2019).

The evidence-based profile is characterized by average to high SES, high motivation (science self-concept), varying cognitive abilities, and an average understanding of the CVS. Students in this profile can be described as being very enthusiastic about science even though they hold strong beliefs in authorities. Correspondingly, this profile is mainly found in younger grades. This group might benefit from hands-on activities to fulfil their desire for experimentation (e.g., Satterthwait, 2010). These experiments should encourage critical thinking about science, for instance, by explicitly reflecting on the approaches that are implemented in class or on interpreting conflicting evidence (e.g., Kienhues et al., 2008). Teaching approaches in which students present and defend their own results from the hands-on phases of experimentation (e.g., the conference method; see Nehring & Lüttgens, 2019) might be very fruitful for incorporating explicit reflections on views into science learning units.

In addition to the relations described in prior theories and studies (see J. A. Chen, 2012; Kampa et al., 2016), we also found some systematic relations for the two “new” types of profiles (mixed, intermediate). Some dimensions of both of these profiles marginally missed the criteria of well-established profiles from prior research. The fact that students in these profiles only partially go in line with the predefined criteria of established profiles might explain the differences in self-concept between these two samples (e.g., Samples 7 & 9). Furthermore, the intermediate profile marginally missed the criteria for being labeled sophisticated. These specific patterns in the profiles let us conclude that students in these profiles might represent intermediate steps that lead from one profile to another. Students in the mixed or intermediate profiles might be located at an intermediate developmental level and might benefit from constructivist and collaborative teaching approaches (Bernholt et al., 2021) as well as a positive epistemic climate (e.g., Muis & Duffy, 2013; Muis et al., 2016).

Taken together, our results highlight comparatively large epistemic diversity within the samples, both in students from the nonacademic track but also from the academic track. In the latter track, we also revealed a range from absolutistic to sophisticated profiles. This diversity of profiles shows that a one-size-fits-all approach to fostering epistemic beliefs is not suitable and that a differentiated or inclusive view is more appropriate. This recently introduced inclusive view welcomes epistemic diversity as a source and not just a burden for science learning (Stinken-Rösner et al., 2020). From this perspective, engaging students from different profiles through collaborative learning (e.g., Heeg et al., 2020) could constitute a fruitful approach for welcoming epistemic diversity. In particular, bringing together students from all profiles might lead to an expression of diverse views that might be particularly valuable for developing epistemic cognition.

### Limitations and Implications for Future Research

In our study, samples from different educational backgrounds and levels were equally weighted. Whereas an argument could be made for different approaches (e.g., balancing profile interpretations by incorporating grades or educational tracks), balancing all samples equally in the integration of results seems to be the most valid and fairest approach for capturing the developmental phases and levels covered by the samples that were included here. Since we integrated more studies from secondary schools than from elementary schools, this approach might have led to an overemphasis of certain characteristics, depending on the research question and focus at hand.

It should be considered that not all covariates were assessed in all the samples (see Fig. 4) and, thus, the picture of the relations between the profiles and external student variables is not complete. However, despite these gaps, our findings extend prior research that looked into relations with covariates (e.g., Kampa et al., 2016) and demonstrate some comparable patterns of relations across samples. Our findings furthermore indicate the importance of taking into account multiple dimensions of epistemic beliefs at the same time when examining their relations with student variables. For example, whereas for students with a multiplistic profile, low self-concept in science was related to a lack of belief in the certainty of scientific knowledge, for students with a sophisticated profile, the same lack of belief was related to high self-concept in science. This demonstrates that the isolated consideration of individual epistemic beliefs might overlook important information.

Our findings have implications for debates regarding the assessment of epistemic beliefs. Despite the different criticisms that have been directed toward Likert-scale questionnaires in recent years (see e.g., Mason, 2016; Sandoval et al., 2016), in our study, this mode of assessment enabled new insights into epistemic beliefs and their relations with student characteristics. Despite differences in item selection and wordings, the Likert-scale questionnaires resulted in rather robust student profiles of epistemic beliefs across the integrated studies. Thus, for the large-scale assessment of epistemic beliefs, Likert-scale questionnaires can deliver reliable insights when combined with person-centered analytic methods. They might however not be well-suited for examining other aspects of epistemic cognition such as epistemic aims, values, and ideals (Barzilai & Chinn, 2018). We obtained these robust results despite differing estimates of internal consistency of the involved scores of epistemic beliefs across the different samples. Particularly in younger samples (e.g., elementary school), internal consistencies appeared to be mediocre (Table S2). Apparently, limited internal consistency does not hinder the informative application of person-centered research on epistemic beliefs. We believe that one reason might be that epistemic beliefs (and profiles thereof) represent formative constructs in the sense of a developmental index, rather than typical reflective latent constructs (Merk & Rosman, 2019). For formative constructs, high internal consistency is neither desirable nor an appropriate indicator of reliability (Stalder et al., 2021; Taber, 2018). To address the criticism that Likert-scale questionnaires ask questions in a manner that is too domain-general (Mason, 2016), in the studies included here, the instrument by Conley et al. (2004) was employed in a domain-specific manner by inserting *science* into the item stems. Even though, by inserting *science*, the instrument still cannot be used to assess epistemic beliefs about specific topics—which may have been advantageous to do for research topics such as epistemic cognition in action (VanSledright & Maggioni, 2016)—covering the more general domain of science offers advantages when comparing learners from different schools or educational contexts. We suggest that researchers employ Likert-scale instruments in a similar manner in future studies by inserting domains or even topics that represent the level of interest into the item stems.

In our study, we undertook *z*-standardization within each sample to build the profiles. This choice directly influences interpretations of the profile labels. For example, in our study, being identified as sophisticated meant that students were sophisticated within their respective school environment. It might seem that the *z*-standardization did not ensure that students receiving this profile label actually showed strong agreement with statements indicating advanced epistemic beliefs across all dimensions. Taking a look at the absolute means of all samples (see profile app; URL blinded to assure a blind review), however, indicated that the profile interpretations remained fairly consistent (e.g., even in the elementary school sample, the sophisticated profile demonstrated values above the scale means in all dimensions). The decision to use *z*-standardization also had the advantage that heterogeneity within a specific context was captured more adequately (Enders & Tofighi, 2007). For example, teachers should know whether some students in their school class show much more advanced epistemic beliefs than their classroom peers so that the teachers can react with individualized educational adaptations.

Our results demonstrate that classifications of profiles of epistemic beliefs have both advantages and disadvantages. An advantage is that person-centered approaches reveal patterns not visible in more regular variable-centered approaches, such as factor analysis and Rasch modeling (Edelsbrunner & Dablander, 2019). The robust findings of such profiles in our study are in line with prior research that has emphasized the advantages of these person-centered models (Schneider & Hardy, 2013; Schwichow et al., 2020). Particularly, our study delivered multiple informative insights regarding the relations with school characteristics and covariates found for the nonlinear as well as overlapping profiles (e.g., the multiplistic & evidence-based profiles). These insights would not be visible in more common statistical models that focus on linear associations between variables (Edelsbrunner & Dablander, 2019). At the same time, however, classification always implies some loss of information. The profiles that received new labels in our study (intermediate, mixed) looked rather similar in shape and level to some of the profile labels that we defined a priori but did not entirely meet their criteria. Furthermore, some of the profiles that received the same label when we applied our criteria differed from each other moderately in the strength and patterns of the four epistemic beliefs. In the case of epistemic beliefs across multiple dimensions, we believe that the advantages clearly outweigh the disadvantages and that person-centered methods for classifying learners provide an informative tool for furthering our understanding of epistemic beliefs.

Finally, we only used cross-sectional data in the present study. An important step forward in harnessing and extending the results will be the implementation of longitudinal studies on the development of epistemic beliefs. Longitudinal studies will allow researchers to gather insights into the developmental nature of the profiles we found. More specifically, it will be possible to observe students’ intraindividual trajectories as they move, for example, from less advanced into more advanced profiles over time and at what stage in development they show intermediate or mixed profile patterns.

### Conclusion

Previous studies had to rely on semantic as well as rather subjective evaluations when comparing their findings with existing studies on epistemic belief profiles. With our study, we have laid a robust empirical foundation for the existence of epistemic belief profiles as well as correlations with external student variables and for differentiated patterns across all grades and tracks. On the one hand, future studies on epistemic beliefs can utilize the rich empirical basis provided by our study to locate their findings not solely within a theoretical framework but also within an empirical framework. On the other hand, our integrative study provides a wide array of insights and of new hypotheses that should be examined in the future. To this end, we have extended the debate about epistemic beliefs in science and given it a broader empirical foundation.

## Supplementary Information

Below is the link to the electronic supplementary material.

Supplementary file1 (DOCX 222 KB)
