# Supplementary material for: Epistemic Beliefs in Science—A Systematic Integration of Evidence From Multiple Studies
Source: Educ Psychol Rev. 2022 Feb 12;34(3):1541–75. doi: 10.1007/s10648-022-09661-w (PMC8853396; doi:10.1007/s10648-022-09661-w)

**Epistemic Beliefs in Science - A Systematic Integration of Evidence From Multiple Studies**

**Supplementary Materials**

| S1: | Descriptions of the Six Studies and the Resulting 12 Samples | 2 |
| --- | --- | --- |
| S2: | Analytic Approach for the Latent Profile Analyses | 5 |
| Table S1: | Overview of Included Studies and Samples | 12 |
| Table S2: | Descriptive Statistics of the Epistemic Belief Dimensions per Sample | 13 |
| Table S3: | Fit Indices of the Latent Profile Analyses Across Samples | 15 |
| Figure S1: | Flowchart of the Systematic Literature Review of Studies That Have Used (a Variation of) the Conley et al. (2004) Questionnaire in German | 19 |

**S1 Descriptions of the Six Studies and the Resulting 12 Samples**

In the following, we describe the six studies and 12 samples stemming from these studies in the order presented in Table S1. We provide information about each study context at the beginning of the description of the first sample for each of the respective studies.

***Study 1: Sample 1; EL3-4I***

The data from Study 1 stemmed from research related to the effectiveness of a state-wide extracurricular enrichment program for elementary school students in southwest Germany, the so-called Hector Children’s Academy Program (HCAP; see Golle et al., 2018). Teachers nominate their students for the program on the basis of their motivation, interest, and school performance. After admission, children can choose from a variety of afternoon STEM courses.

The data from Sample 1 (EL3-4I) stemmed from regular elementary school classes (see Schiefer et al., 2019). All students from these classes took part in the study, and no selection procedure was involved. It included 456 elementary students in Grades 3 and 4 (52% boys; Grade 3: 252, Grade 4: 200, Grade not known: 5) and consisted of 42 classes from 10 public elementary schools in urban areas in southwest Germany. The aim of this study was to evaluate a new instrument for assessing elementary school students’ understanding of the *scientific inquiry cycle* (SIC) that was afterwards used to evaluate the effectiveness of some courses in the HCAP (e.g., Schiefer et al., 2017; Schiefer, Stark, et al., 2020).

***Study 1: Sample 2; EL3-4II***

The students in Sample 2 (EL3-4II) were participants in the HCAP enrichment program. The data derived from a series of intervention studies in this program that investigated the effectiveness of STEM courses at the HCAP on the development of students’ epistemic beliefs (Schiefer et al., 2017; Schiefer, Golle, et al., 2020; Schiefer, Stark, et al., 2020). The sample comprised 680 elementary school students (65% boys; Grade 3: 347, Grade 4: 328, Grade not known: 5). Participants were mostly from middle-class families living in urban areas in southwest Germany. Data about ethnicity were not collected. For the present analyses, data from the pretest measures before the intervention were used.

***Study 2: Sample 3; MS5-6***

Study 2 was an intervention study aimed at supporting the development of epistemic beliefs (Walkowiak & Nehring, 2018). The sample included 151 students in the sixth (*n* = 98) and seventh grades (*n* = 50 students; *n* = 3 missing) from mixed-track secondary schools (which do not differentiate between the academic & nonacademic tracks). Half of the students worked on a German version of the assessment instrument by Conley et al. (2004). The other half received a substantially modified version and were therefore not included in the current study. For the present analyses, data from the pretest were used.

***Study 3: Sample 4; NAS7-9***

Study 3 examined relations between students’ science-related epistemic beliefs and their perceptions about teaching and classroom characteristics in science. A total of 954 secondary school students from all tracks were assessed. Sample 4 included 335 students in Grades 7 to 9 (46% boys; Grade 7: 81, Grade 8: 102, Grade 9: 138) from nonacademic track secondary schools.

***Study 3: Sample 5; AS7-9I***

This sample consisted of 619 students (47% boys) from the academic track (*Gymnasium*) in Grades 7 to 9 (Grade 7: 195, Grade 8: 102, Grade 9: 275).

***Study 4: Sample 6; AS5-6***

The data from Study 4 were based on the binational project Development of Learning in Science (DoLiS; Bernholt et al., 2021) in which secondary school students were investigated in Sweden and Germany in a cross-sectional, two-cohort longitudinal design. For the current study, data from the German sample of 2,977 students in Grades 5 to 12 from 164 classes in eight secondary schools in northern Germany were used. The students were asked to fill out paper-pencil questionnaires comprising items about students’ family background, motivation, interest, classroom characteristics, and epistemic beliefs as well as an achievement test in chemistry in their school setting. The majority of the students (91%) were enrolled in the academic track. Sample 6 included 887 fifth and sixth graders (49% boys; Grade 5: 574, Grade 6: 313).

***Study 4: Sample 7; AS7-9II***

This sample comprised 1,146 secondary school students in Grades 7 to 9 (47% boys; Grade 7: 284, Grade 8: 296, Grade 9: 539) from the DoLiS study.

***Study 4: Sample 8; AS10-12I***

Sample 8 consisted of a total of 990 students in Grades 10 to 12 (41% boys), with 350 tenth graders, 500 eleventh graders, and 121 twelfth graders from the DoLiS study.

***Study 5: Sample 9; NAS10***

The samples in Study 5 stemmed from a large-scale study carried out in eight federal states by the Institute for Educational Quality Improvement at Humboldt University (IQB) in 2009. The aim of the study was to pilot items for educational standards in science (see Lenski et al., 2016; Pant et al., 2013). The sample consisted of 2,558 tenth graders (50% boys) who were enrolled in either nonacademic track secondary schools (*Real-, Mittel-, Sekundarschule*) or mixed-track secondary schools (*Gesamtschule,* general qualification for university entrance is possible) and answered a questionnaire comprising the items on epistemic beliefs in science.

***Study 5: Sample 10; AS9***

This sample from the same IQB study consisted of 2,437 tenth graders (46% boys) enrolled in academic track secondary schools (*Gymnasium*). On average, 17 students per class (*SD* = 4.60) received the questionnaire that included scientific epistemic belief items.

***Study 6: Sample 11; AS8-9***

The cross-sectional Study 6 assessed views on the nature of scientific inquiry (Nehring, 2020; Reith & Nehring, 2020), inquiry competences, and content knowledge in the contexts of both biology and chemistry in 794 eighth to 12th graders (Schwichow & Nehring, 2018) from academic track schools in two federal states in Germany. Sample 11 comprised 364 students in the eighth (*n* = 24) and ninth grades (*n* = 340).

***Study 6: Sample 12; AS10-12II***

This sample included 430 students in the 10^th^ (*n* = 226), 11^th^ (*n* = 191), and 12^th^ grades (*n* = 13).

**S2 Analytic Approach for the Latent Profile Analyses**

We followed the common stepwise strategy used in latent profile analysis (S. L. Ferguson et al., 2020; Hickendorff et al., 2018; Oberski, 2016). First, we ran latent profile analyses based on the four scale means of epistemic beliefs with increasing numbers of profiles in each data set. We started with one profile and increased successively to six profiles. Then, we examined the relative fits of the models and chose the preferred model with the respective number of profiles in each sample. Various measures of relative model fit can be examined in order to evaluate the different profile solutions from a latent profile analysis. We mainly interpreted two model fit criteria: The Bayesian Information Criterion (BIC) and the Luong-Vo-Mendell-Rubin Likelihood Ratio Test (VL-LRT), both of which have been shown to provide preferable indices of model fit in simulation studies (Asparouhov & Muthén, 2012; Nylund et al., 2007). According to these criteria, a model is preferred if it shows a lower BIC than the other models and if it shows the largest number of latent profiles while still maintaining a significant VL-LRT (Hickendorff et al., 2018). We did not focus on additional indices such as the AIC/aBIC, further likelihood ratio tests, entropy, or a threshold for the percentage of students in the smallest profile (Hickendorff et al., 2018). AIC/aBIC and further likelihood ratio tests tend to be less reliable than the BIC for selecting the best model (Asparouhov & Muthén, 2012; Nylund et al., 2007), entropy does not indicate model fit but instead indicates reliability (Collins & Lanza, 2010), and the size of the smallest profile was not of central interest to us because, according to our research aims, we also wanted to extract profiles with potentially small numbers of students, as long as the fit indices indicated that these were reliably present.

After selecting the preferred model in each data set, we examined and interpreted the resulting profiles on the basis of our a priori criteria for profile classifications. This led to a theoretically grounded label for each of the resulting student profiles of epistemic beliefs. Finally, on the basis of the bias-corrected hypothesis testing (*BCH*) procedure, the currently most reliable method for relating latent profiles to covariates (Asparouhov & Muthén, 2014), we added covariates to the model in each data set to examine the extent to which membership in each of the profiles was correlated with external student characteristics.

In creating the latent profiles, missing data were handled by employing full information maximum likelihood (FIML) estimation in Mplus (Muthén & Muthén, 1998-2017). \\Because FIML cannot handle missing data in covariates, we applied multiple imputation for the covariate analyses, with 50 imputations for each sample.

References

Asparouhov, T., & Muthén, B. O. (2012). Using Mplus TECH11 and TECH14 to test the number of latent classes. *Mplus Web Notes*, *14*, 1–17. https://www.statmodel.com/examples/webnotes/webnote14.pdf

Asparouhov, T., & Muthén, B. O. (2014). Auxiliary variables in mixture modeling: Three-step approaches using Mplus. *Structural Equation Modeling*, *21*(3), 329–341. https://doi.org/10.1080/10705511.2014.915181

Bernholt, A., Lindfors, M., & Winberg, M. (2019). Students’ epistemic beliefs in Sweden and Germany and their interrelations with classroom characteristics. *Scandinavian Journal of Educational Research*, 1–17. https://doi.org/10.1080/00313831.2019.1651763

Collins, L. M., & Lanza, S. T. (2010). *Latent class and latent transition analysis: With applications in the social, behavioral, and health sciences* (718th ed.). John Wiley & Sons.

Conley, A. M., Pintrich, P. R., Vekiri, I., & Harrison, D. (2004). Changes in epistemological beliefs in elementary science students. *Contemporary Educational Psychology*, *29*(2), 186–204. https://doi.org/10.1016/j.cedpsych.2004.01.004

Ferguson, S. L., Moore, E. W. G., & Hull, D. M. (2020). Finding latent groups in observed data: A primer on latent profile analysis in Mplus for applied researchers. *International Journal of Behavioral Development*, *44*(5), 458–468. https://doi.org/https://doi.org/10.1177/0165025419881721

Golle, J., Zettler, I., Rose, N., Trautwein, U., Hasselhorn, M., & Nagengast, B. (2018). Effectiveness of a “grass roots” statewide enrichment program for gifted elementary school children. *Journal of Research on Educational Effectiveness*, *11*(3), 1–34. https://doi.org/10.1080/19345747.2017.1402396

Hickendorff, M., Edelsbrunner, P. A., McMullen, J., & Schneider, M. (2018). Informative tools for characterizing individual differences in learning: Latent class, latent profile, and latent transition analysis. *Learning and Individual Differences*, *66*, 4–15. https://doi.org/10.1016/j.lindif.2017.11.001

Lenski, A. E., Hecht, M., Penk, C., Milles, F., Mezger, M., Heitmann, P., Stanat, P., & Pant, H. A. (2016). IQB-Ländervergleich 2012. Skalenhandbuch zur Dokumentation der Erhebungsinstrumente [IQB cross-country comparison 2012. Documentation of assessment instruments]. https://doi.org/10.20386/HUB-42547

Muthén, B. O., & Muthén, L. K. (2017). *Mplus user’s guide (8th ed.)*. Muthén & Muthén.

Nehring, A. (2020). Naïve and informed views on the nature of scientific inquiry in large‐scale assessments: Two sides of the same coin or different currencies? *Journal of Research in Science Teaching, 57*(4), 510–535. https://doi.org/10.1002/tea.21598

Nylund, K. L., Asparouhov, T., & Muthén, B. O. (2007). Deciding on the number of classes in latent class analysis and growth mixture modeling: A Monte Carlo simulation study. *Structural Equation Modeling*, *14*(4), 535–569. http://www.tandfonline.com/doi/abs/10.1080/10705510701575396%0Apapers3://publication/uuid/69D03B5F-89D8-4DF4-95E5-3B4235C79C2C

Oberski, D. (2016). Mixture models: Latent profile and latent class analysis. In J. Robertson & M. Kaptein (Eds.), *Modern statistical methods for HCI. Human-computer interaction series* (pp. 275–287). Springer.

Pant, H. A., Stanat, P., Schroeders, U., Roppelt, A., Siegle, T., & Pöhlmann, C. (2013). IQB-Ländervergleich 2012. Mathematische und naturwissenschaftliche Kompetenzen am Ende der Sekundarstufe I [IQB cross-national comparison 2012. Mathematical and science competencies at the end of secondary school level]. Waxmann.

Reith, M., & Nehring, A. (2020). Scientific reasoning and views on the nature of scientific inquiry: Testing a new framework to understand and model epistemic cognition in science. *International Journal of Science Education, 42*(16), 2716–2741. https://doi.org/https://doi.org/10.1080/09500693.2020.1834168

Schiefer, J., Golle, J., Tibus, M., & Oschatz, K. (2019). Scientific reasoning in elementary school children: Assessment of the inquiry cycle. *Journal of Advanced Academics*, *30*(2), 144–177. https://doi.org/10.1177/1932202X18825152

Schiefer, J., Golle, J., Tibus, M., Herbein, E., Gindele, V., Trautwein, U., & Oschatz, K. (2020). Effects of an extracurricular science intervention on elementary school children’s epistemic beliefs – A randomized controlled trial. *British Journal of Educational Psychology*, *90*, 382–402. https://doi.org/10.1111/bjep.12301

Schiefer, J., Stark, L., Gaspard, H., Wille, E., Trautwein, U., & Golle, J. (2020). Scaling up an extracurricular science intervention for elementary school students: It works, and girls benefit more from it than boys. *Journal of Educational Psychology*, Advance Online Publication. https://doi.org/10.1037/edu0000630

Schwichow, M., & Nehring, A. (2018). Variablenkontrolle beim Experimentieren in Biologie, Chemie und Physik: Höhere Kompetenzausprägungen bei der Anwendung der Variablenkontrollstrategie durch höheres Fachwissen? Empirische Belege aus zwei Studien [Control of variables in experiments]. *Zeitschrift Für Didaktik Der Naturwissenschaften*, *24*, 271–233. https://doi.org/https://doi.org/10.1007/s40573-018-0085-8

**Table S1**

*Overview of Included Studies and Samples*

| Study | Sample # | Sample label | School type | Grade level | *N* | Age  *M* (*SD*) |
| --- | --- | --- | --- | --- | --- | --- |
| 1 | 1 | EL3-4I | Elementary | 3-4 | 456 | 8.85 (0.77) |
|  | 2 | EL3-4II^a^ | Elementary enrichment | 3-4 | 680 | 8.78 (0.72) |
| 2 | 3 | MS5-6 | Secondary mixed track | 5-6 | 151 | 12.17 (0.73) |
| 3 | 4 | NAS7-9 | Secondary nonacademic track | 7-9 | 277 | - |
|  | 5 | AS7-9I | Secondary academic track | 7-9 | 604 | - |
| 4 | 6 | AS5-6 | Secondary academic track | 5-6 | 868 | 10.98 (0.75) |
|  | 7 | AS7-9II | Secondary academic track | 7-9 | 1126 | 13.85 (1.03) |
|  | 8 | AS10-12I | Secondary academic track | 10-12 | 979 | 16.77 (1.11) |
| 5 | 9 | NAS10 | Secondary nonacademic track | 10 | 2558 | 15.60 (0.82) |
|  | 10 | AS10 | Secondary academic track | 10 | 2437 | 15.35 (0.59) |
| 6 | 11 | AS8-9 | Secondary academic track | 8-9 | 364 | 14.53 (0.77) |
|  | 12 | AS10-12II | Secondary academic track | 10-12 | 430 | 16.18 (0.93) |

*Note*. Sample labels: EL = elementary schools, AS = academic track secondary schools, NAS = nonacademic track secondary schools, numbers indicate the range of the grade levels within the respective sample, Roman numerals I and II added to prevent duplicate labels. *-* = no data for this grade level group.

^a^ Elementary Enrichment: data were collected in a sample of students who participated in an extracurricular STEM enrichment program.

**Table S2**

*Descriptive Statistics of the Epistemic Belief Dimensions per Sample*

| Sample label | Likert scale | Dimension | # items | *M (SD)* | *α* |
| --- | --- | --- | --- | --- | --- |
| ELS3-4I  (*n* = 456) | 1-4 | Source | 5 | 2.43 (0.60) | .59 |
|  |  | Certainty | 5 | 2.46 (0.57) | .58 |
|  |  | Development | 7 | 3.12 (0.53) | .57 |
|  |  | Justification | 9 | 3.40 (0.41) | .65 |
| EL3-4II  (*n* = 680) | 1-4 | Source | 5 | 2.58 (0.64) | .72 |
|  |  | Certainty | 5 | 2.62 (0.60) | .68 |
|  |  | Development | 7 | 3.31 (0.46) | .62 |
|  |  | Justification | 9 | 3.50 (0.33) | .60 |
| MS5-6  (*n* = 151) | 1-4 | Source | 5 | 3.29 (0.72) | .68 |
|  |  | Certainty | 5 | 3.43 (0.68) | .79 |
|  |  | Development | 5 | 3.71 (0.59) | .82 |
|  |  | Justification | 5 | 3.77 (0.53) | .64 |
| NAS7-9  (*n* = 335) | 1-6 | Source | 5 | 3.19 (0.97) | .82 |
|  |  | Certainty | 5 | 3.89 (0.99) | .81 |
|  |  | Development | 5 | 3.27 (1.02) | .86 |
|  |  | Justification | 5 | 3.46 (0.96) | .81 |
| AS7-9I  (*n* = 619) | 1-6 | Source | 5 | 2.97 (0.85) | .76 |
|  |  | Certainty | 5 | 4.17 (0.98) | .79 |
|  |  | Development | 5 | 3.74 (0.82) | .83 |
|  |  | Justification | 5 | 4.04 (0.72) | .75 |
| AS5-6  (*n* = 887) | 1-5 | Source | 5 | 3.30 (0.85) | .75 |
|  |  | Certainty | 4 | 3.08 (0.81) | .57 |
|  |  | Development | 4 | 3.71 (0.78) | .62 |
|  |  | Justification | 4 | 3.83 (0.75) | .61 |
| AS7-9II  (*n* = 1146) | 1-5 | Source | 5 | 3.42 (0.76) | .70 |
|  |  | Certainty | 4 | 3.47 (0.75) | .60 |
|  |  | Development | 4 | 3.84 (0.74) | .68 |
|  |  | Justification | 4 | 3.84 (0.69) | .62 |
| AS10-12I  (*n* = 990) | 1-5 | Source | 5 | 3.64 (0.71) | .60 |
|  |  | Certainty | 4 | 3.79 (0.67) | .72 |
|  |  | Development | 4 | 3.95 (0.65) | .65 |
|  |  | Justification | 4 | 3.81 (0.63) | .62 |
| NAS10  (*n* = 2558) | 1-4 | Source | 5 | 2.74 (0.61) | .67 |
|  |  | Certainty | 7 | 2.74 (0.58) | .76 |
|  |  | Development | 8 | 3.10 (0.48) | .79 |
|  |  | Justification | 8 | 3.14 (0.46) | .76 |
| AS10  (*n* = 2437) | 1-4 | Source | 5 | 3.06 (0.60) | .69 |
|  |  | Certainty | 7 | 3.02 (0.55) | .76 |
|  |  | Development | 8 | 3.32 (0.45) | .80 |
|  |  | Justification | 8 | 3.28 (0.40) | .72 |
| AS8-9  (*n* = 364) | 1-4 | Source | 5 | 3.26 (0.62) | .79 |
|  |  | Certainty | 7 | 3.30 (0.57) | .76 |
|  |  | Development | 8 | 3.40 (0.54) | .82 |
|  |  | Justification | 7 | 3.41 (0.50) | .75 |
| AS10-12II  (*n* = 430) | 1-4 | Source | 5 | 3.31 (0.54) | .76 |
|  |  | Certainty | 5 | 3.47 (0.48) | .73 |
|  |  | Development | 5 | 3.53 (0.48) | .81 |
|  |  | Justification | 5 | 3.53 (0.44) | .75 |

**Table S3**

*Fit Indices of the Latent Profile Analyses Across Samples*

|  | | | Information criteria | | | Likelihood ratio tests | | |  |  |
| --- | --- | --- | --- | --- | --- | --- | --- | --- | --- | --- |
| Profile | Loglikelihood | #Parameters | *AIC* | *BIC* | *aBIC* | *p* LMR | *p* VL-LRT | *p* BLRT | Entropy | ø class assignment probability |
| 1 | -1,389 | 8 | 2,794 | 2,827 | 2,801 | NA | NA | NA | NA | NA |
| 2 | -1,309 | 17 | 2,651 | 2,721 | 2,667 | <.001 | <.001 | <.001 | .58 | .87/.89 |
| 3 | -1,250 | 26 | 2,552 | 2,659 | 2,577 | <.001 | <.001 | <.001 | .65 | .84/.87/.85 |
| **4** | **-1,217** | **35** | **2,503** | **2,647** | **2,536** | **.058** | **.055** | **<.001** | **.69** | **.82/.85/.81/.85** |
| 5 | -1,201 | 44 | 2,489 | 2,671 | 2,531 | .444 | .439 | .100 | .72 | .80/.84/.8/.97/.82 |
| 6 | -1,186 | 53 | 2,478 | 2,696 | 2,528 | .576 | .572 | .044 | .77 | .80/.98/.81/.84/.81/.87 |
| 1 | -1,389 | 8 | 2,794 | 2,827 | 2,801 | NA | NA | NA | NA | NA |
| 2 | -1,781 | 17 | 3,597 | 3,674 | 3,620 | <.001 | <.001 | <.001 | .65 | .92/.86 |
| **3** | **-1,686** | **26** | **3,424** | **3,541** | **3,459** | **.003** | **.003** | **<.001** | **.70** | **.90/.84/.86** |
| 4 | -1,641 | 35 | 3,352 | 3.51 | 3,399 | .328 | .323 | <.001 | .68 | .82/.79/.86/.79 |
| 5 | -1,600 | 44 | 3,289 | 3,487 | 3,348 | .109 | .106 | <.001 | .74 | .96/.84/.80/.84/.82 |
| 6 | -1,578 | 53 | 3,263 | 3,502 | 3,334 | .127 | <.001 | .002 | .77 | .74/.78/.84/.83/.96/.82 |
| 1 | -570 | 8 | 1,157 | 1,181 | 1,155 | NA | NA | NA | NA | NA |
| 2 | -534 | 17 | 1,102 | 1,154 | 1,100 | .010 | .010 | <.001 | .92 | .99/.93 |
| 3 | -512 | 26 | 1,076 | 1,154 | 1,072 | .565 | .567 | .006 | .90 | .97/.93/.89 |
| **4** | **-492** | **35** | **1,055** | **1,160** | **1,050** | **.012** | **.012** | **.024** | **.93** | **.97/.92/.96/.98** |
| 5 | -479 | 44 | 1,047 | 1,180 | 1,040 | .166 | .159 | .229 | .92 | .97/.86/.94/.97/.94 |
| 6 | -468 | 53 | 1,043 | 1,203 | 1,035 | .055 | .052 | .341 | .92 | .97/.97/.95/.87/.92/.93 |
| 1 | -1,536 | 8 | 3,089 | 3,118 | 3,092 | NA | NA | NA | NA | NA |
| 2 | -1,431 | 17 | 2,896 | 2,957 | 2,904 | .038 | .036 | <.001 | .76 | .92/.93 |
| **3** | **-1,369** | **26** | **2,790** | **2,884** | **2,802** | **.020** | **.018** | **<.001** | **.80** | **.90/.93/.91** |
| 4 | -1,332 | 35 | 2,733 | 2,860 | 2,749 | .109 | .105 | <.001 | .85 | .90/.94/.90/.95 |
| 5 | -1,303 | 44 | 2,693 | 2,853 | 2,713. | .043 | .040 | .002 | .87 | .93/.85/.95/.93/.91 |
| 6 | -1,286 | 53 | 2,678 | 2,870 | 2,702 | .522 | .514 | .161 | .81 | .81/.93/.90/.82/.91/.92 |
| 1 | -2,943 | 8 | 5,902 | 5,937 | 5,911 | NA | NA | NA | NA | NA |
| 2 | -2,772 | 17 | 5,577 | 5,652 | 5,598 | <.001 | <.001 | <.001 | .70 | .94/.87 |
| **3** | **-2,644** | **26** | **5,339** | **5,454** | **5,371** | **<.001** | **<.001** | **<.001** | **.73** | **.90/.88/.88** |
| 4 | -2,599 | 35 | 5,269 | 5,423 | 5,312 | .277 | .271 | <.001 | .71 | .90/.84/.81/.85 |
| 5 | -2,558 | 44 | 5,204 | 5,398 | 5,258 | - | - | - | .70 | .78/.89/.78/.84/.82 |
| 6 | -2,531 | 53 | 5,169 | 5,402 | 5,234 | .189 | .184 | <.001 | .71 | .90/.78/.79/.81/.80/.80 |
| 1 | -4,121 | 8 | 8,258 | 8,296 | 8,270 | NA | NA | NA | NA | NA |
| 2 | -3,971 | 17 | 7,976 | 8,057 | 8,003 | .001 | <.001 | <.001 | .61 | .88/.88 |
| 3 | -3,852 | 26 | 7,757 | 7,881 | 7,798 | .069 | .066 | <.001 | .67 | .85/.85/.84 |
| **4** | **-3,775** | **35** | **7,620** | **7,787** | **7,676** | **.097** | **.094** | **<.001** | **.72** | **.84/.86/.83/.84** |
| 5 | -3,739 | 44 | 7,566 | 7,776 | 7,636 | .074 | .072 | <.001 | .77 | .89/.85/.85/.86/.86 |
| 6 | -3,726 | 53 | 7,558 | 7,811 | 7,642 | .554 | .552 | .574 | .76 | .86/.90/.84/.85/.78/.85 |
| 1 | -4,995 | 8 | 10,006 | 10,047 | 10,021 | NA | NA | NA | NA | NA |
| 2 | -4,776 | 17 | 9,586 | 9,671 | 9,617 | <.001 | <.001 | <.001 | .71 | .84/.93 |
| 3 | -4,665 | 26 | 9,382 | 9,512 | 9,430 | .032 | .030 | <.001 | .61 | .82/.83/.82 |
| **4** | **-4,100** | **35** | **8,270** | **8,446** | **8,335** | **-** | **-** | **-** | **.73** | **.73/.89/.83/1.00** |
| 5 | -4,535 | 44 | 9,159 | 9,380 | 9,240 | - | - | - | .74 | .85/.83/.82/.82/.77 |
| 6 | -4,502 | 53 | 9,111 | 9,377 | 9,209 | .285 | .282 | <.001 | .72 | .81/.78/.79/.79/.80/.80 |
| 1 | -3,958 | 8 | 7,932 | 7,971 | 7,946 | NA | NA | NA | NA | NA |
| 2 | -3,757 | 17 | 7,548 | 7,631 | 7,577 | <.001 | <.001 | <.001 | .62 | .92/.84 |
| 3 | -3,666 | 26 | 7,383 | 7,510 | 7,428 | .001 | .001 | <.001 | .63 | .86/.81/.81 |
| **4** | **-3,609** | **35** | **7,287** | **7,458** | **7,347** | **.018** | **.017** | **<.001** | **.67** | **.86/.81/.78/.82** |
| 5 | -3.580 | 44 | 7,248 | 7,463 | 7,323 | .155 | .151 | <.001 | .67 | .81/.75/.80/.77/.81 |
| 6 | -3,557 | 53 | 7,221 | 7,480 | 7,311 | .136 | .132 | .006 | .68 | .82/.76/.74/.78/.77/.70 |
| 1 | -7,941 | 8 | 15,898 | 15,577 | 15,551 | NA | NA | NA | NA | NA |
| 2 | -7,198 | 17 | 14,429 | 14,529 | 14,475 | <.001 | <.001 | <.001 | .65 | .87/.91 |
| 3 | -6,641 | 26 | 13,333 | 13,485 | 13,403 | <.001 | <.001 | <.001 | .78 | .93/.89/.9 |
| 4 | -6,171 | 35 | 12,423 | 12,628 | 12,517 | <.001 | <.001 | <.001 | .80 | .94/.89/.87/.91 |
| 5 | -6,017 | 44 | 12,122 | 12,379 | 12,240 | .019 | .019 | <.001 | .77 | .88/.94/.82/.81/.89 |
| **6** | **-5,863** | **53** | **11,833** | **12,142** | **11,974** | **<.001** | **<.001** | **<.001** | **.77** | **.94/.81/.84/.85/.82/.88** |
| 1 | -6,933 | 8 | 13,882 | 13,928 | 13,903 | NA | NA | NA | NA | NA |
| 2 | -6,097 | 17 | 12,228 | 12,326 | 12,272 | <.001 | <.001 | <.001 | .66 | .92/.88 |
| **3** | **-5,804** | **26** | **11,660** | **11,811** | **11,729** | **<.001** | **<.001** | **<.001** | **.77** | **.91/.90/.89** |
| 4 | -5,505 | 35 | 11,079 | 11,282 | 11,171 | <.001 | <.001 | <.001 | .77 | .92/.81/.88/.88 |
| 5 | -5,323 | 44 | 10,734 | 10,989 | 10,849 | .139 | .136 | <.001 | .75 | .81/.91/.86/.83/.84 |
| 6 | -5,237 | 53 | 10,581 | 10,888 | 10,720 | .002 | .002 | <.001 | .77 | .99/.91/.79/.86/.83/.85 |
| 1 | -1,196 | 8 | 2,409 | 2,440 | 2,415 | NA | NA | NA | NA | NA |
| 2 | -959 | 17 | 1,952 | 2,018 | 1,964 | .002 | .002 | <.001 | .88 | .98/.94 |
| **3** | **-858** | **26** | **1,768** | **1,869** | **1,787** | **.688** | **.684** | **<.001** | **.82** | **.92/.91/.94** |
| 4 | -844 | 35 | 1,758 | 1,894 | 1,783 | .312 | .313 | .294 | .81 | .89/.89/.76/.91 |
| 5 | -832 | 44 | 1,753 | 1,924 | 1,784 | .024 | .023 | .275 | .85 | .97/.89/.72/.93/.90 |
| 6 | -832 | 53 | 1,771 | 1,977 | 1,809 | .240 | .240 | .496 | .87 | .89/.97/.93/.72/.90/1.00 |
| 1 | -1,183 | 8 | 2,382 | 2,415 | 2,383 | NA | NA | NA | NA | NA |
| 2 | -869 | 17 | 1,771 | 1,841 | 1,773 | .087 | .084 | <.001 | .84 | .97/.95 |
| **3** | **-767** | **26** | **1,586** | **1,691** | **1,589** | **<.001** | **<.001** | **<.001** | **.79** | **.91/.90/.91** |
| 4 | -748 | 35 | 1,566 | 1,708 | 1,597 | .234 | .229 | .036 | .82 | .84/.87/.91/.92 |
| 5 | -757 | 44 | 1,603 | 1,782 | 1,613 | .330 | .328 | .333 | .85 | .91/1.00/.91/.90/1.00 |
| 6 | -857 | 53 | 1,819 | 2,034 | 1,866 | .240 | .240 | 1.00 | .94 | 1.00/1.00/.97/.94/1.00/1.00 |

*Note*. *AIC* = Akaike information criterion; *BIC* = Bayesian information criterion; *aBIC* = sample-size adjusted Bayesian information criterion; *p LMR* = *p*-value from Lo-Mendell-Rubin adjusted likelihood ratio test; *p VL-LRT* = p-value from Vuong-Lo-Mendell-Rubin likelihood ratio test; *p BLRT* = *p*-value from bootstrapped likelihood ratio test; *Groups below 5%* indicates the profile probability estimate for the smallest profile if the respective model showed a profile with a size below 5%. In the models in Study 2, for Sample 4 with four and five profiles, a few variances had to be fixed in order to enable the model to converge; likelihood ratio tests were not available in these cases. NA = For solutions with one profile, no likelihood ratio tests, Entropy, or average class assignment probabilities are provided because these indices are not meaningful when there are fewer than two profiles. Selected models are marked in bold.

**Figure S1**

*Flowchart of the Systematic Literature Review of Studies That Have Used (a Variation of) the Conley et al. (2004) Questionnaire in German*


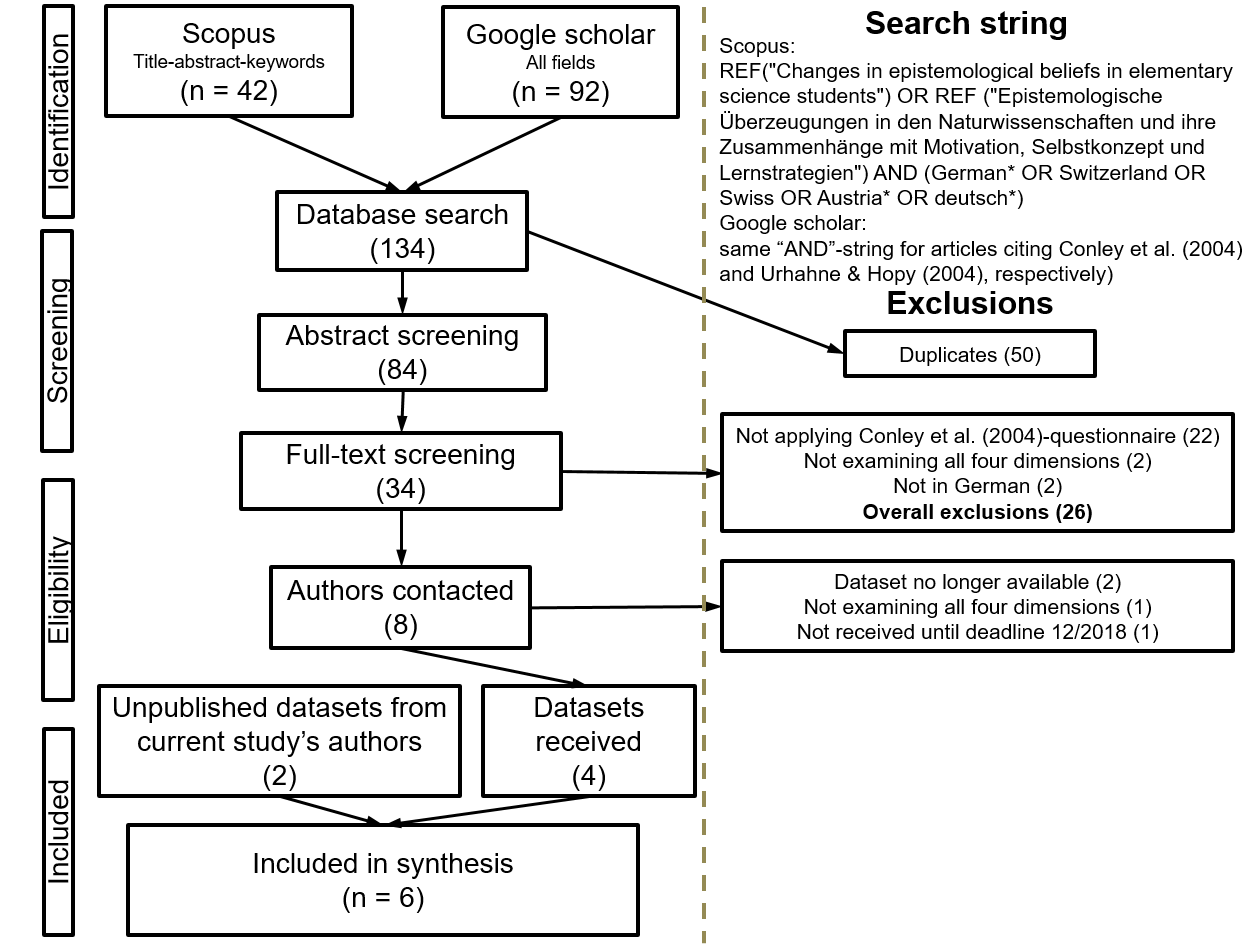

Supplement: Supplementary file 1 — Supplementary file1 (DOCX 222 KB) [file 10648_2022_9661_MOESM1_ESM.docx]
